# Beyond the trigger: the unknown precipitant as a high-risk phenotype in acute heart failure

**DOI:** 10.3389/fcvm.2026.1880740

**Published:** 2026-10-30

**Authors:** Georgios Aletras, Maria Bachlitzanaki, Maria Stratinaki, Emmanuel Lamprogiannakis, Emmanuel Foukarakis, Yannis Pantazis, Michalis Hamilos, Kostas Stylianou

**Affiliations:** 1 Department of Cardiology, Venizelio General Hospital of Heraklion, Heraklion, Greece; 2 School of Medicine, University of Crete, Heraklion, Greece; 3 Second Department of Internal Medicine, Venizelio General Hospital of Heraklion, Heraklion, Greece; 4 Institution of Applied and Computational Mathematics, Foundation of Research and Technology-Hellas, Heraklion, Greece; 5 Department of Cardiology, University General Hospital of Heraklion, Heraklion, Greece; 6 Department of Nephrology, University General Hospital of Heraklion, Heraklion, Greece

**Keywords:** acute heart failure, cardiorenal syndrome, chronic kidney disease, frailty, medication non-compliance, precipitating factors, right ventricular dysfunction, unknown precipitant

## Abstract

**Background:**

A substantial proportion of patients with acute heart failure (AHF) present without an identifiable precipitating factor. We compared the clinical profile, echocardiographic characteristics, and mid-term outcomes of AHF patients stratified by precipitating factor category.

**Methods:**

We conducted a prospective single-center observational study at Venizelio General Hospital, Heraklion, including 486 consecutive patients hospitalized with AHF between February 2023 and January 2025. Patients were classified into three groups: behavioral (*n* = 148), non-behavioral/clinical (*n* = 230), and unknown (*n* = 108). The primary endpoint was all-cause death at 12 months; secondary endpoints were terminal renal replacement therapy (RRT) and a composite of death, AHF rehospitalization or RRT. Groups were compared by Kaplan–Meier analysis and Cox regression with the behavioral group as reference, and the cumulative incidence of first AHF rehospitalization was estimated with death and terminal RRT as competing events.

**Results:**

The unknown group was older and more frail (CFS ≥5), with more advanced chronic kidney disease, more right heart failure and lower TAPSE, higher NT-proBNP and lower admission eGFR. All-cause mortality at 12 months was 41.7% versus 20.3% and 29.6% (log-rank *p* < 0.001; unknown vs. behavioral HR 2.44, 95% CI 1.54–3.88), with a parallel gradient for the composite endpoint (event-free survival 35.2% vs. 58.8% and 52.6%; HR 1.96, 1.39–2.77). After adjustment for age, admission eGFR, right heart failure, NT-proBNP and frailty, precipitant category no longer improved model fit (likelihood ratio *p* = 0.066) and the association of the unknown group with mortality was attenuated and no longer significant (adjusted HR 1.43, 0.88–2.31). With death and terminal RRT as competing events, 12-month cumulative incidence of first AHF rehospitalization did not differ (36.1% vs. 29.7% and 26.1%; *p* = 0.207). ACEi/ARB prescription at discharge was lower in the unknown group (21.2% vs. 39.0% and 44.2%, *p* < 0.001).

**Conclusions:**

In AHF, the absence of an identifiable precipitating factor identifies a high-risk phenotype characterized by advanced multimorbidity, right ventricular dysfunction, renal impairment and worse mid-term outcomes. The difference was driven predominantly by mortality rather than rehospitalization, and the association was attenuated after adjustment for the phenotype-defining variables. The label may therefore serve as an early bedside marker of advanced disease, requiring confirmation in multicenter cohorts.

## 1 Introduction

Acute heart failure (AHF) represents one of the leading causes of emergency hospitalization worldwide, carrying substantial short- and long-term morbidity and mortality (1, 2). Despite significant advances in the pharmacological and device-based management of chronic heart failure (HF), the burden of AHF-related hospitalizations has remained largely unchanged over the past two decades, underscoring the critical importance of understanding the mechanisms that drive decompensation (1, 3). Notably, the contemporary AHF population is aging rapidly, with the majority of patients now presenting beyond the eighth decade of life — a shift that reflects the success of preventive and therapeutic cardiology, but that also demands recognition that biological age alone should not exclusively dictate therapeutic decision-making.

We hypothesized that patients with no identifiable precipitating factor represent a distinct high-risk subgroup in whom decompensation reflects the natural trajectory of advanced, multimorbid, end-stage HF rather than a discrete triggering event.

## 2 Methods

### 2.1 Study design and population

This was a prospective observational study conducted at the Cardiology Department of Venizelio General Hospital, Heraklion, Crete, Greece. Data from consecutive patients admitted between February 2023 and January 2025 with a primary diagnosis of AHF were collected. Data were recorded prospectively at the point of care into a dedicated study database, using standardized admission and discharge forms completed by the treating cardiologist; the electronic hospital information system and structured follow-up contacts were used to complete outcome ascertainment. AHF diagnosis required the co-existence of clinical signs and symptoms (dyspnea, orthopnea, elevated jugular venous pressure, lower limb edema, pulmonary crackles on auscultation) together with objective laboratory and imaging findings (elevated natriuretic peptides, evidence of structural cardiac disease, dilated inferior vena cava, presence of B-lines on lung ultrasound), in accordance with the 2021 European Society of Cardiology (ESC) Heart Failure Guidelines (1). Patients under 18 years of age, those already on renal replacement therapy (RRT) and those hospitalized primarily for ST-elevation myocardial infarction (STEMI) or acute coronary syndrome without a dominant AHF presentation were excluded. All participants provided written informed consent and the study was approved by the Scientific Council of Venizelio General Hospital of Heraklion on 5 August 2022 (protocol number 15/05-08-2022). Within this cohort, the objective was to test the hypothesis that patients without an identifiable precipitant constitute a distinct high-risk phenotype, by comparing the clinical characteristics, echocardiographic profile, and mid-term outcomes of AHF patients stratified by precipitating factor category, with particular focus on the unknown group.

### 2.2 Data collection

At admission, baseline demographic characteristics (age, sex), primary HF etiology (ischemic, valvular, hypertensive, or other), functional status using the New York Heart Association (NYHA) classification, and a comprehensive comorbidity profile were recorded. Comorbidities documented included coronary artery disease (CAD), arterial hypertension, atrial fibrillation (AF), chronic kidney disease (CKD), diabetes mellitus, active malignancy, airway disease, stroke, and active smoking. Admission laboratory values included creatinine (Cr), estimated glomerular filtration rate (eGFR), albumin, potassium, sodium, urea, C-reactive protein (CRP), N-terminal pro-B-type natriuretic peptide (NT-proBNP), high-sensitivity troponin I (hs-cTnI), serum glutamic-oxaloacetic transaminase (SGOT), serum glutamic-pyruvic transaminase (SGPT), red cell distribution width (RDW), hemoglobin, and white blood cell count (WBC). Maximum in-hospital creatinine and minimum eGFR were also recorded. Chloride and urinary albumin-to-creatinine ratio (UACR) were not available for all patients, as their systematic measurement was incorporated into the protocol following an amendment during the study period (chloride: *n* = 353; UACR: *n* = 270).

The precipitating factor of each decompensation episode was documented prospectively and in real time by the treating cardiologist at the time of admission, as a mandatory field of the departmental admission protocol, before any study-related data extraction took place. The study investigators did not assign or reassign precipitants retrospectively; their role was limited to mapping the prospectively documented precipitant onto the three pre-specified categories. Accordingly, the “unknown” designation reflects the absence of an identifiable trigger after structured bedside assessment by the admitting cardiologist, rather than a failure of documentation.

Follow-up was completed to 12 months for the entire cohort through outpatient clinic visits or structured telephone contact, supplemented by the hospital information system.

### 2.3 Definitions

Right heart failure (RHF) was defined by the co-existence of at least two of the following three domains: (i) clinical signs of systemic venous congestion — at least one of: elevated jugular venous pressure, peripheral edema, hepatomegaly, or ascites — in the absence of a predominant left-sided etiology; (ii) echocardiographic morphological and functional criteria — right ventricular (RV) dilation [RV/left ventricular (LV) basal diameter ratio >1.0 or qualitative RV enlargement] and/or RV systolic dysfunction (tricuspid annular plane systolic excursion [TAPSE] < 17 mm and/or tricuspid lateral annular systolic velocity [TV S'] < 9.5 cm/s); and (iii) echocardiographic evidence of elevated right-sided pressures — estimated pulmonary artery systolic pressure (PASP) > 35 mmHg or moderate-to-high echocardiographic probability of pulmonary hypertension (PH) as defined in Supplementary Table S1. Isolated RHF was defined as RHF occurring in the absence of significant LV dysfunction.

CAD was defined as the presence of ≥50% stenosis in at least one epicardial vessel on coronary angiography or documented equivalent non-invasive evidence.

CKD was defined as eGFR <60 mL/min/1.73m^2^ persisting for ≥3 months, and was staged according to the Kidney Disease: Improving Global Outcomes (KDIGO) 2012 classification (Supplementary Table S2). Baseline renal function was defined from outpatient serum creatinine values recorded within the 12 months preceding the index admission, in the absence of intercurrent acute illness; where several qualifying values were available, the value judged most representative of the patient's steady state was used. For patients without a qualifying pre-admission measurement, the creatinine level at which the patient stabilized during the index hospitalization was taken as the baseline, and the presence of acute kidney injury was adjudicated retrospectively against that value. Baseline eGFR was calculated from the baseline creatinine using the CKD-EPI equation and was available for all 486 patients.

Frailty was assessed using the Clinical Frailty Scale (CFS) applied to the patient's baseline functional status prior to the acute decompensation. Patients were categorized as: non-frail (CFS 1–3), pre-frail/mildly frail (CFS 4–5), and frail (CFS 6–9) (Supplementary Table S3).

PH probability was classified as low, moderate, or high based on estimated PASP (ePASP = 4v^2^ + RAP, where v is the peak tricuspid regurgitation velocity measured by continuous-wave Doppler, and RAP is right atrial pressure, estimated from inferior vena cava dimensions and collapsibility) combined with additional indirect echocardiographic indicators (Supplementary Table S1). In cases of severe tricuspid regurgitation with low peak velocity — not representative of the true degree of PH — ePASP was deliberately excluded to avoid underestimation.

Clinical phenotypes at presentation were classified according to the 2021 ESC HF Guidelines as: acute decompensated heart failure (ADHF), acute pulmonary edema (APO), isolated RHF, and cardiogenic shock (CS) (Supplementary Table S4).

Multivalvular disease was defined as the presence of at least moderate regurgitation or stenosis in more than one cardiac valve, as graded by the echocardiographer according to current ESC/European Association of Cardiovascular Imaging (EACVI) guidelines.

### 2.4 Echocardiographic assessment

Transthoracic echocardiographic studies were performed during hospitalization and interpreted by experienced cardiologists. LV ejection fraction (LVEF) was measured using Simpson's biplane method and categorized as preserved (≥50%), mildly reduced (41%–49%), or reduced (≤40%). Valvular heart disease and its severity were documented. RV systolic function was assessed by TV S' velocity and TAPSE. LV diastolic function was evaluated by the E/e′ ratio. Left atrial volume index (LAVi) was measured and indexed to body surface area. Estimated PASP (ePASP) was calculated as described above.

### 2.5 Classification of precipitating factors

For each patient, a systematic attempt was made to identify the probable cause(s) of decompensation. The diagnostic work-up informing this assessment was uniform across the cohort and independent of HF chronicity: the departmental admission protocol mandated 12-lead electrocardiography, chest radiography, bedside lung and cardiac ultrasound, admission natriuretic peptide and high-sensitivity troponin measurement and a full biochemical and inflammatory panel for every patient, with microbiological sampling whenever infection was clinically suspected. No structured per-patient checklist of candidate precipitants was completed, so the identification of a precipitant rested on the admitting cardiologist's judgment applied to this common set of investigations, with the two investigators subsequently mapping the documented precipitant onto the three pre-specified categories (Section 2.2). Since multiple concurrent precipitants were frequently identified, patients were classified into three mutually exclusive categories based on the predominant factor:

#### 2.5.1 Behavioral

Non-compliance with dietary recommendations (excess sodium or fluid intake, consumption of processed foods, alcohol misuse — distinct from chronic alcoholism) or non-compliance with prescribed pharmacological therapy (partial or complete).

Behavioral precipitants were not restricted to patients with previously established HF. In *de novo* presentations, a behavioral precipitant was assigned only when the admitting physician judged, on structured history taking, that dietary or lifestyle factors had played a decisive role in unmasking a pre-existing but previously undiagnosed structural substrate — for example, high habitual salt intake and uncontrolled blood pressure in a patient with previously unrecognized mitral regurgitation. Acute ischemia presenting as APO was classified as a clinical, not behavioral, precipitant.

#### 2.5.2 Non-behavioral/Clinical

One or more of the following: (i) uncontrolled hypertension/hypertensive surge; (ii) concurrent infection, diagnosed by clinical examination, elevated inflammatory markers, or imaging findings; (iii) arrhythmia (rapid AF, ventricular tachycardia, or hemodynamically significant bradyarrhythmia); (iv) drug toxicity (chronic alcoholism, recent initiation of potentially cardiotoxic chemotherapy or immunotherapy in the absence of other clear precipitants); and (v) acute ischemia, defined by significant elevation of myocardial injury biomarkers, new regional wall motion abnormalities in a coronary distribution, or concurrent ischemic electrocardiographic (ECG) changes — applicable only to non-ST-elevation myocardial infarction (NSTEMI) presentations with a dominant AHF picture.

#### 2.5.3 Unknown

No precipitating factor identifiable after systematic assessment at admission. This category was reserved exclusively for patients in whom no trigger could be identified; patients with an identified but atypical trigger were assigned to the non-behavioral/clinical group according to the nature of that trigger. In this cohort no patient was classified here on the basis of an unclassifiable trigger: all 108 had zero precipitating factors recorded (Table 5).

When behavioral and clinical precipitants co-existed, the patient was assigned to the category judged to have the greater contribution. In cases of ambiguity or approximately equal contribution, patients were assigned to the behavioral group, as one of the study objectives was also to evaluate the prognostic impact of patient adherence and lifestyle habits. Two or more concurrent precipitants were identified in 157 patients (32.3%); in 84 of these a behavioral and a clinical trigger co-existed, and 75 were assigned to the behavioral group while nine were assigned to the non-behavioral/clinical group because the clinical trigger was judged predominant (Table 5).

### 2.6 Follow-up and endpoints

Patients were followed for 12 months through outpatient clinic visits or structured telephone contact. The primary endpoint was all-cause death; the secondary endpoint was need for RRT. Emergency-department (ED) visits, all-cause and AHF-specific rehospitalizations, and a composite endpoint of death, AHF rehospitalization or RRT were also recorded. A further composite of death or terminal RRT, referred to throughout as the death/RRT composite, was analysed alongside these. Death and RRT were treated as cumulative events ascertained over the full cohort; for the composite endpoints a patient reaching RRT was counted as an event irrespective of subsequent death, and death without prior RRT was not counted as an RRT event; for the secondary endpoint of terminal RRT, dialysis begun during the index hospitalization in a patient who died before a permanent modality could be established was recorded as an RRT episode but not as an endpoint event, that patient contributing to the mortality endpoint instead. The RRT endpoint denotes terminal RRT, that is, dialysis initiated and maintained as a permanent modality; a dialysis episode followed by recovery of renal function — whether occurring during the index hospitalization or after discharge — was recorded as an RRT episode but was not counted as an RRT endpoint or as a component of the composite. Three patients met this description: one during the index hospitalization and two after discharge.

Non-fatal recurrent events (rehospitalization, ED visits) were assessed among patients alive and free of RRT during the relevant interval, as those reaching the primary or secondary endpoint were no longer at risk. These non-fatal events were ascertained only in patients who remained alive and free of terminal RRT, so events occurring in patients who subsequently died were not captured; the proportions reported for individual non-fatal endpoints therefore refer to that denominator and are not first-event rates. The triple composite, by contrast, was defined in the whole cohort as the first occurrence of death, AHF rehospitalization or RRT. To address the resulting bias, the cumulative incidence of first AHF rehospitalization was additionally estimated with death and terminal RRT as competing events using the Aalen–Johansen estimator, the order of events being determined by comparing the time to the first composite event with the time to death or terminal RRT, both recorded in weeks, so that a patient whose first composite event preceded death or RRT was classified as having been rehospitalized first. This recovered 53 first AHF rehospitalizations absent from the survivor-conditional follow-up field because the patient had by then reached a competing event and left the survivor-based denominator (51 death, two terminal RRT); groups were compared by Gray's test and by a Fine–Gray subdistribution model. Mortality was ascertained for the entire cohort. In-hospital deaths were additionally adjudicated as cardiovascular or non-cardiovascular by two cardiologists from the complete clinical record; cause of death could not be reliably adjudicated for deaths occurring after discharge, and post-discharge mortality is therefore reported as all-cause only.

### 2.7 Statistical analysis

Continuous variables are presented as median (25th–75th percentile), following confirmation of non-normal distribution by the Shapiro–Wilk test. Categorical variables are presented as frequencies and percentages. Between-group comparisons used the Kruskal–Wallis test for continuous variables and the chi-square test for categorical variables. Kaplan–Meier survival curves were constructed for the triple composite endpoint, all-cause mortality, and the death/RRT composite. Differences between survival curves were assessed by the log-rank test, with pairwise comparisons performed to identify which group pairs drove overall significance. Unadjusted Cox proportional hazards regression was performed for each time-to-event endpoint (triple composite, all-cause mortality, and the death/RRT composite) to quantify between-group hazard ratios (HRs) with 95% confidence intervals (CIs), using the behavioral group as the reference category. The proportional hazards assumption was verified using Schoenfeld residuals and was satisfied in all primary models; the single exception, which arose in one of the renal sensitivity models, is reported in Section 3.8. Multivariable Cox models were constructed for each endpoint, including age, admission eGFR, RHF, log-transformed NT-proBNP and baseline frailty (CFS ≥5). Because these variables are constitutive features of the unknown-precipitant phenotype rather than external confounders, the multivariable models were not interpreted as estimates of an independent causal effect of precipitant category. Instead, a likelihood ratio test comparing nested models with and without precipitant category was used to determine whether the categorization carries prognostic information incremental to the measured phenotype-defining variables; discrimination was summarized by Harrell's C-index. Two pre-specified sensitivity analyses were performed: the first repeated the Kaplan–Meier analysis after excluding patients who died during the index hospitalization, to confirm that the prognostic separation represented a post-discharge phenomenon; the second repeated all survival analyses after excluding patients with *de novo* HF, in whom behavioral precipitants operate on a different baseline. A further sensitivity analysis addressed the renal component of the phenotype: the unadjusted and full multivariable models for all-cause death were refitted substituting baseline eGFR, or baseline KDIGO stage 3B–4, for admission eGFR, and with both renal measures entered together, in order to separate the contribution of CKD from that of the change in renal function at presentation. A two-tailed *p*-value <0.05 was considered statistically significant. Statistical analyses were performed using IBM SPSS Statistics version 25.0 (IBM Corp., Armonk, NY, USA) and Python version 3.12.3 (Python Software Foundation, https://www.python.org/). Cox proportional hazards regression and Kaplan–Meier survival analyses were performed using the lifelines library (version 0.30.3). Data management and descriptive statistics used the pandas (version 2.3.3), NumPy (version 2.4.4), and SciPy (version 1.17.1) libraries; competing-risk analyses used scikit-survival (version 0.28.0); figures were generated with Matplotlib (version 3.10.8).

## 3 Results

### 3.1 Study population and precipitating factor distribution

A total of 486 patients with AHF were enrolled over a 24-month period (February 2023 – January 2025). Baseline characteristics of the overall cohort are summarized in Tables 1–4. The median age was 81 years and 53.5% were male. *De novo* HF accounted for 27.2% of presentations. The most prevalent primary etiology was ischemic cardiomyopathy (32.9%), followed by other/unknown (23.9%), valvular (22.4%), and hypertensive (20.8%). A history of valve replacement was present in 6.2% of patients (aortic 4.9%, mitral 1.0%, combined 0.2%). CAD was confirmed in 46.3%, while in 32.5% the coronary status was undefined. Implantable cardioverter-defibrillator (ICD) and permanent pacemaker (PPM) were implanted in 5.6% and 9.3% respectively, and 11.9% had undergone prior coronary artery bypass grafting (CABG) (Table 1).

**Table 1:** Baseline general characteristics, comorbidities of the total study cohort (*n* = 486).

Parameter	Number (%)
Demographics:	
Male sex	260 (53.5%)
*de novo* HF	132 (27.2%)
Age (years)	81 (74−86)
Primary Etiology:	
Ischemic	160 (32.9%)
Valvular	109 (22.4%)
Hypertensive	101 (20.8%)
Other/Unknown	116 (23.9%)
History of valve replacement:	
Aortic	24 (4.9%)
Mitral	5 (1%)
Aortic+Mitral	1 (0.2%)
Multivalvular disease	163 (33.5%)
Previous HF hospitalization within 2y	138 (28.4%)
Atrial Fibrillation:	
Paroxysmal	90 (18.5%)
Chronic	174 (35.8%)
Unknown	26 (5.3%)
Coronary artery disease:	
No	103 (21.2%)
Yes	225 (46.3%)
Undefined	158 (32.5%)
Other comorbidities:	
ICD	27 (5.6%)
PPM	45 (9.3%)
CABG	58 (11.9%)
Arterial Hypertension	424 (87.2%)
Diabetes mellitus	240 (49.4%)
Airway disease	152 (31.3%)
CKD	261 (53.7%)
Active malignancy	48 (9.9%)
Active smoking	51 (10.5%)
Right HF	210 (43.2%)
Stroke:	
Ischemic	38 (7.8%)
Hemorrhagic	1 (0.2%)

Values are presented as *n* (%) unless otherwise stated.

HF, heart failure; ADHF, acute decompensated heart failure; CKD, chronic kidney disease; AF, atrial fibrillation; CAD, coronary artery disease; ICD, implantable cardioverter defibrillator; PPM, permanent pacemaker; CABG, coronary artery bypass grafting.

**Table 2:** Clinical phenotype, CKD staging (KDIGO), functional status (NYHA, clinical frailty scale), urinary albumin-to-creatinine ratio category, and pre-admission/discharge pharmacological therapy of the total study cohort (*n* = 486). Values are presented as *n* (%). Discharge medication was recorded in survivors of the index hospitalization (*n* = 460).

Parameter	Number (%)
Clinical phenotype:	
ADHF	361 (74.3%)
Acute pulmonary edema	117 (24.1%)
Isolated right HF	2 (0.4%)
Cardiogenic shock	6 (1.2%)
Chronic HF medication:	
ARNI	20 (4.1%)
B-blocker	281 (57.8%)
MRA	107 (22%)
SGLT2i	98 (20.2%)
Furosemide	251 (51.6%)
ACEi/ARBs	239 (49.2%)
CKD stage (KDIGO):	
1	44 (9.1%)
2	181 (37.2%)
3A	124 (25.5%)
3B	101 (20.8%)
4	36 (7.4%)
Baseline NYHA:	
I	19 (3.9%)
II	91 (18.7%)
III	296 (60.9%)
IV	80 (16.5%)
Baseline CFS:	
CFS 1–3	40 (8.2%)
CFS 4–5	270 (55.6%)
CFS 6–9	176 (36.2%)
Baseline CFS (Binary):	
CFS ≤4	161 (33.1%)
CFS ≥5	325 (66.9%)
UACR category (*n* = 270):	
<30 mg/g Cr	67 (24.8%)
30–299 mg/g Cr	132 (48.9%)
>300 mg/g Cr	71 (26.3%)
Discharge HF medication (*n* = 460):	
ARNIs	50 (10.9%)
B-blockers	360 (78.3%)
MRAs	272 (59.1%)
ACEi/ARBs	173 (37.6%)
SGLT2i	294 (63.9%)
Furosemide	410 (89.1%)

ADHF, acute decompensated heart failure; HF, heart failure; KDIGO, Kidney Disease: Improving Global Outcomes; NYHA, New York Heart Association; CFS, clinical frailty scale; UACR, urinary albumin-to-creatinine ratio; ARNI, angiotensin receptor–neprilysin inhibitor; MRA, mineralocorticoid receptor antagonist; SGLT2i, sodium-glucose cotransporter-2 inhibitor; ACEi, angiotensin-converting enzyme inhibitor; ARB, angiotensin receptor blocker.

Post-discharge non-fatal outcomes were ascertained among patients alive and free of terminal renal replacement therapy at each time point.

**Table 3:** Echocardiographic characteristics of the total study cohort (*n* = 486).

Echocardiographic parameter	Number (%)
LV dilation	122 (25.1%)
RV dilation	121 (24.9%)
LVEF:	
≥50%	239 (49.2%)
41%–49%	41 (8.4%)
≤40%	206 (42.4%)
Tricuspid regurgitation:	
No/Mild	271 (55.7%)
Moderate	118 (24.3%)
Severe	97 (20%)
Mitral regurgitation:	
No/Mild	276 (56.8%)
Moderate	162 (33.3%)
Severe	48 (9.9%)
Mitral stenosis:	
No/Mild	464 (95.5%)
Moderate	17 (3.5%)
Severe	5 (1%)
Aortic stenosis:	
No/Mild	385 (79.2%)
Moderate	39 (8%)
Severe	62 (12.8%)
Aortic regurgitation:	
No/Mild	424 (87.2%)
Moderate	56 (11.5%)
Severe	6 (1.2%)
Probability of PH:	
Low	67 (13.8%)
Moderate	164 (33.7%)
High	255 (52.5%)

Values are presented as *n* (%) unless otherwise stated. LVEF, left ventricular ejection fraction; LV, left ventricle; RV, right ventricle; PH, pulmonary hypertension. LVEF was measured by Simpson's biplane method and categorized as preserved (≥50%), mildly reduced (41%–49%), or reduced (≤40%). Valvular disease severity was graded as no/mild, moderate, or severe according to current echocardiographic guidelines. PH probability was classified as low, moderate, or high based on estimated pulmonary artery systolic pressure and additional indirect echocardiographic signs, as described in Section 2.3 and Supplementary Table S1.

**Table 4:** Admission laboratory values and echocardiographic quantitative parameters for the total study cohort (*n* = 486).

Parameter	Median (25th – 75th percentile)
Systolic BP (mmHg)	145 (126–170)
eGFR (mL/min/1.73 m**^2^**)	56 (40–75)
Cr (mg/dl)	1.14 (0.92–1.54)
Baseline Cr (mg/dl)	1.1 (0.9–1.4)
Baseline eGFR (mL/min/1.73 m**^2^**)	57 (43–76)
Maximum Cr (mg/dl)	1.47 (1.12–2)
Minimum eGFR (mL/min/1.73 m**^2^**)	43 (28–59)
Urea (mg/dl)	56 (43–81)
Albumin (g/dl)	4 (3.8–4.3)
WBC (×10⁹/L)	8.65 (6.7–11.1)
CRP (mg/dl)	0.9 (0.4–2.2)
SGOT (IU/L)	27 (21–39)
SGPT (IU/L)	19 (13–32)
RDW (%)	16.2 (15–18.3)
NT-proBNP (pg/mL)	5294 (3112–10613)
Hemoglobin (g/dl)	11.7 (10.4–13)
Hs-Troponin-I (pg/mL)	28.77 (13.95–68.95)
Na (mEq/L)	139 (137–141)
K (mEq/L)	4.52 (4.16–4.91)
Cl (mEq/L) [*n* = 353]	102 (99.7–105.5)
UACR (mg/g Cr) [*n* = 270]	105.96 (30.04–329.17)
Echocardiographic values:	
PASP (mmHg)	40 (35–55)
E/e′	16.5 (13–23)
TV S**’** (cm/s)	10 (9–12)
LAVi (mL/m**^2^**)	51 (43–63)
LVEF (%)	45 (30–55)
TAPSE (mm)	17 (15–19)
Hospital days (*n* = 460)	7 (5–9)

Values are presented as median (25th–75th percentile). BP, blood pressure; eGFR, estimated glomerular filtration rate (CKD-EPI formula); Cr, creatinine; WBC, white blood cell count; CRP, C-reactive protein; RDW, red cell distribution width; NT-proBNP, N-terminal pro-B-type natriuretic peptide; hs-cTnI, high-sensitivity cardiac troponin I; SGOT, serum glutamic-oxaloacetic transaminase; SGPT, serum glutamic-pyruvic transaminase; Na, sodium; K, potassium; Cl,  chloride; UACR, urinary albumin-to-creatinine ratio; PASP, pulmonary artery systolic pressure; E/e′, ratio of mitral inflow early diastolic velocity to mitral annular early diastolic velocity; TV S’, tricuspid lateral annular systolic velocity; LAVi, left atrial volume index indexed to body surface area; LVEF, left ventricular ejection fraction; TAPSE, tricuspid annular plane systolic excursion.

The comorbidity burden was substantial. Arterial hypertension was present in 87.2%, diabetes mellitus in 49.4%, CKD in 53.7%, and airway disease in 31.3%. KDIGO staging of the whole cohort revealed that 25.5% were stage G3a, 20.8% stage G3b and 7.4% stage G4; among the 261 patients with CKD these correspond to 47.5%, 38.7% and 13.8% respectively. AF was highly prevalent: 18.5% had paroxysmal AF, 35.8% chronic AF, and 5.3% had AF of undetermined type. Ischemic stroke was documented in 7.8% and hemorrhagic stroke in 0.2%. Active malignancy was present in 9.9% (Tables 1, 2).

Baseline functional status, assessed prior to the acute decompensation episode, demonstrated a high burden of frailty. The majority of patients were in NYHA class III (60.9%) or IV (16.5%) at baseline, and only 3.9% were in class I. Using the CFS, 36.2% were classified as frail (CFS 6–9), 55.6% as pre-frail (CFS 4–5), and only 8.2% as non-frail (CFS 1–3). Overall, 66.9% had a CFS score of 5 or above, corresponding to at least mild frailty on the native CFS (Table 2).

Pre-admission HF pharmacotherapy is detailed in Table 2. Beta-blockers were the most commonly prescribed agent (57.8%), followed by furosemide (51.6%), angiotensin-converting enzyme inhibitors/angiotensin receptor blockers (ACEi/ARBs; 49.2%), mineralocorticoid receptor antagonists (MRAs; 22.0%), sodium-glucose cotransporter-2 inhibitors (SGLT2i; 20.2%), and angiotensin receptor–neprilysin inhibitors (ARNIs; 4.1%). At discharge (*n* = 460, excluding in-hospital deaths), loop diuretic prescription was near-universal (89.1%), with beta-blocker uptake of 78.3%, MRAs 59.1%, SGLT2i 63.9%, ACEi/ARBs 37.6%, and ARNIs 10.9%, reflecting the drive toward pharmacological optimization during hospitalization (Table 2).

The predominant clinical phenotype at presentation was ADHF (74.3%), followed by APO (24.1%), CS (1.2%), and isolated RHF (0.4%). RHF, as defined in Section 2.3, was present in 43.2% of the overall cohort. Multivalvular disease was documented in 33.5% (Tables 1, 2).

Echocardiographic findings for the total cohort are presented in Table 3. LVEF was preserved (≥50%) in 49.2%, mildly reduced (41%–49%) in 8.4%, and reduced (≤40%) in 42.4%. The most frequent valvular abnormality was tricuspid regurgitation (moderate 24.3%, severe 20.0%), followed by mitral regurgitation (moderate 33.3%, severe 9.9%), aortic stenosis (moderate 8.0%, severe 12.8%), and aortic regurgitation (moderate 11.5%, severe 1.2%). LV dilation was present in 25.1% and RV dilation in 24.9%. PH was classified as high probability in 52.5%, moderate in 33.7%, and low in 13.8% (Table 3).

Key quantitative laboratory and echocardiographic parameters are shown in Table 4. Median admission eGFR was 56 mL/min/1.73m^2^, with a median creatinine of 1.14 mg/dl. NT-proBNP was markedly elevated at a median of 5294 pg/mL. Median hemoglobin was 11.7 g/dl, reflecting the high prevalence of anemia in this elderly, multimorbid population. Among the 270 patients (55.6%) in whom UACR was measured, 48.9% had microalbuminuria (30–299 mg/g Cr), 26.3% macroalbuminuria (>300 mg/g Cr), and 24.8% had a normal UACR (<30 mg/g Cr). Median LVEF was 45%, TAPSE 17 mm, TV S' 10 cm/s, PASP 40 mmHg, E/e′ 16.5, and LAVi 51 mL/m^2^. Median hospital length of stay was 7 days (Table 4).

Regarding precipitating factors (Table 5), dietary non-compliance was the most frequently identified trigger (28.2%), followed by uncontrolled hypertension (23.3%), arrhythmia (21.8%), infection (19.3%), medication non-compliance (15.4%), acute ischemia (10.3%), and drug toxicity (0.6%). No identifiable precipitant was found in 22.2% of patients. Multiple concurrent precipitating factors were common: 45.5% of patients had a single precipitant, 24.7% had two, and a further 7.6% had three or more (3 factors: 6.6%; 4 factors: 0.8%; 5 factors: 0.2%). In total, 157 patients (32.3%) carried two or more concurrent triggers, illustrating the complex pathophysiology of AHF in this population. Patients were ultimately classified as behavioral (*n* = 148, 30.5%), non-behavioral/clinical (*n* = 230, 47.3%), and unknown (*n* = 108, 22.2%).

**Table 5:** Distribution of precipitating factors and number of concurrent triggers in the total study cohort (*n* = 486).

Parameter	Number (%)
Non-compliance with dietary limitations	137 (28.2%)
Non-compliance with medication	75 (15.4%)
Uncontrolled BP	113 (23.3%)
Acute ischemia	50 (10.3%)
Arrhythmia	106 (21.8%)
Infection	94 (19.3%)
Toxic agents	3 (0.6%)
Unknown	108 (22.2%)
Number of precipitating factors:	
0	108 (22.2%)
1	221 (45.5%)
2	120 (24.7%)
3	32 (6.6%)
4	4 (0.8%)
5	1 (0.2%)

Values are presented as *n* (%). BP, blood pressure. The precipitating factor was documented prospectively at admission by the treating cardiologist, as a mandatory field of the departmental admission protocol; two investigators subsequently mapped each documented precipitant onto the three pre-specified categories, resolving ambiguous cases by consensus (Section 2.2). Patients may have had more than one concurrent precipitating factor; therefore, the sum of individual factor frequencies exceeds 100%. The number of precipitating factors is the count of individual triggers recorded for each patient and is derived directly from the individual factor fields listed above, with patients in the “0” category corresponding to the unknown group. Patients were ultimately classified into three mutually exclusive groups based on the predominant precipitant: behavioral (non-compliance with dietary recommendations or pharmacological therapy; *n* = 148, 30.5%), non-behavioral/clinical (arrhythmia, infection, uncontrolled hypertension, acute ischemia, or drug toxicity; *n* = 230, 47.3%), and unknown (no precipitating factor identifiable after systematic assessment; *n* = 108, 22.2%).

A summary of all endpoints is provided in Table 6. During the index hospitalization, 26 patients (5.3%) died (cardiovascular death 3.9%) and 12 (2.5%) required RRT. Over the 12-month follow-up, all-cause mortality was 29.4% (143 patients), with death occurring in 11.9% at 3 months and 18.3% at 6 months. The triple composite endpoint (death, AHF rehospitalization, or RRT) was reached by 36.8% at 6 months and 49.4% at 12 months. Follow-up endpoints beyond the primary outcome were recorded in survivors not requiring RRT, with denominators decreasing over time due to attrition from death and RRT initiation (*n* = 421 at 3 months, *n* = 386 at 6 months, *n* = 335 at 12 months). Among these surviving patients, cumulative rates of ED visits rose progressively from 41.8% at 3 months to 64.2% at 12 months, and AHF-specific rehospitalization from 14.3% to 26.9%.

**Table 6:** In-hospital outcomes, primary and secondary endpoints, and follow-up events at 3, 6, and 12 months for the total study cohort.

Endpoints	Number (%)
Triple endpoint (6M)	179 (36.8%)
Triple endpoint (12M)	240 (49.4%)
In hospital:	
RRT	12 (2.5%)
All-cause death	26 (5.3%)
Cardiovascular death	19 (3.9%)
Primary endpoint:	
Death (3M)	58 (11.9%)
Death (6M)	89 (18.3%)
Death (12M)	143 (29.4%)
Secondary endpoint:	
Terminal RRT by 3 months (*n* = 486)	8 (1.6%)
Terminal RRT by 6 months (*n* = 486)	10 (2.1%)
Terminal RRT by 12 months (*n* = 486)	11 (2.3%)
Rest endpoints:	
3 months (*n* = 421):	
ED visit (any cause)	176 (41.8%)
Rehospitalization (any cause)	118 (28.0%)
Rehospitalization (for AHF)	60 (14.3%)
6 months (*n* = 386):	
ED visit (any cause)	206 (53.4%)
Rehospitalization (any cause)	143 (37.0%)
Rehospitalization (for AHF)	79 (20.5%)
12 months (*n* = 335):	
ED visit (any cause)	215 (64.2%)
Rehospitalization (any cause)	155 (46.3%)
Rehospitalization (for AHF)	90 (26.9%)

Values are presented as *n* (%). RRT, renal replacement therapy; AHF, acute heart failure; ED, emergency department. All-cause death was the primary endpoint. Need for RRT was a secondary endpoint. Follow-up events (ED visits, rehospitalizations) were recorded only in patients who survived the index hospitalization and did not require RRT; the denominator therefore decreases over time due to attrition from death and RRT initiation. The denominator at each time point is the number of patients with a recorded assessment and differs slightly from the number free of death and terminal RRT at that time: four assessments were not recorded (three at 6 months and one at 12 months) and two were recorded in the interval in which the patient reached an endpoint (one at 3 months and one at 6 months) (3 months: *n* = 421; 6 months: *n* = 386; 12 months: *n* = 335). The triple composite endpoint comprises all-cause death, AHF rehospitalization, or renal replacement therapy.

### 3.2 Clinical characteristics by precipitating factor group

The three groups differed significantly across multiple clinical dimensions. The unknown group was significantly older (83 vs. 80 and 80 years; *p* = 0.018), more frail (CFS 6–9: 53.7% vs. 33.1% behavioral and 30.0% non-behavioral; *p* = 0.001), and had a higher proportion of patients with CFS ≥5 (78.7% vs. 63.5% in both other groups; *p* = 0.012). Advanced CKD (stage 3B–4) was substantially more prevalent (46.3% vs. 23.6% and 22.6%; *p* < 0.001), as was the overall CKD burden (69.4% vs. 49.3% and 49.1%; *p* = 0.001). RHF was disproportionately represented in the unknown group (66.7% vs. 44.6% and 31.3%; *p* < 0.001), as was multivalvular disease (47.2% vs. 27.0% and 31.3%; *p* = 0.002). A valvular etiology was significantly more common (32.4% vs. 18.2% and 20.4%), while hypertensive etiology was strikingly rare (8.3% vs. 25.0% and 23.9%; *p* = 0.009). Arterial hypertension as a comorbidity was correspondingly less prevalent (72.2% vs. 92.6% and 90.9%; *p* < 0.001). *De novo* HF was least frequent in the unknown group (13.9% vs. 22.3% and 36.5%; *p* < 0.001). Chronic persistent/permanent AF was highest in the unknown group (52.8% vs. 36.5% and 27.4%), and NYHA class IV at baseline was more than twice as common (29.6% vs. 13.5% and 12.2%; *p* < 0.001) (Tables 7, 8).

**Table 7:** Baseline general characteristics and comorbidities stratified by precipitating factor group.

Parameter	Behavioral (*n* = 148)	Non-behavioral (*n* = 230)	Unknown (*n* = 108)	*P*-value
Demographics:				
Male sex	83 (56.1%)	114 (49.6%)	63 (58.3%)	0.241
*de novo* HF	33 (22.3%)	84 (36.5%)	15 (13.9%)	<0.001
Age (years)	80 [72.5–85]	80 [73–86]	83 [77–87.5]	0.018
Primary Etiology:				0.009
Ischemic	50 (33.8%)	73 (31.7%)	37 (34.3%)	
Valvular	27 (18.2%)	47 (20.4%)	35 (32.4%)	
Hypertensive	37 (25.0%)	55 (23.9%)	9 (8.3%)	
Other/Unknown	34 (23.0%)	55 (23.9%)	27 (25.0%)	
Right HF	66 (44.6%)	72 (31.3%)	72 (66.7%)	<0.001
History of valve replacement:				0.205
Aortic	10 (6.8%)	6 (2.6%)	8 (7.4%)	
Mitral	1 (0.7%)	4 (1.7%)	0 (0%)	
Aortic+Mitral	0 (0%)	1 (0.4%)	0 (0%)	
Multivalvular disease	40 (27.0%)	72 (31.3%)	51 (47.2%)	0.002
Previous HF hospitalization within 2y	36 (24.3%)	67 (29.1%)	35 (32.4%)	0.159
Atrial Fibrillation:				<0.001
Paroxysmal	27 (18.2%)	54 (23.5%)	9 (8.3%)	
Chronic	54 (36.5%)	63 (27.4%)	57 (52.8%)	
Unknown	4 (2.7%)	20 (8.7%)	2 (1.9%)	
Coronary artery disease:				0.525
No	25 (16.9%)	55 (23.9%)	23 (21.3%)	
Yes	69 (46.6%)	105 (45.7%)	51 (47.2%)	
Undefined	54 (36.5%)	70 (30.4%)	34 (31.5%)	
Other comorbidities:				
ICD	7 (4.7%)	12 (5.2%)	8 (7.4%)	0.622
PPM	17 (11.5%)	13 (5.7%)	15 (13.9%)	0.027
CABG	19 (12.8%)	21 (9.1%)	18 (16.7%)	0.126
Arterial Hypertension	137 (92.6%)	209 (90.9%)	78 (72.2%)	<0.001
Diabetes mellitus	74 (50%)	109 (47.4%)	57 (52.8%)	0.642
Airway disease	51 (34.5%)	74 (32.2%)	27 (25%)	0.251
CKD	73 (49.3%)	113 (49.1%)	75 (69.4%)	0.001
Active malignancy	10 (6.8%)	25 (10.9%)	13 (12.0%)	0.295
Active smoking	20 (13.5%)	25 (10.9%)	6 (5.6%)	0.118
Stroke:				0.875
Ischemic	12 (8.1%)	17 (7.4%)	9 (8.3%)	
Hemorrhagic	0 (0%)	1 (0.4%)	0 (0%)	

Values are presented as *n* (%). Between-group comparisons were performed using the chi-square test. HF, heart failure; AF, atrial fibrillation; CAD, coronary artery disease; ICD, implantable cardioverter defibrillator; PPM, permanent pacemaker; CABG, coronary artery bypass grafting; CKD, chronic kidney disease; RHF, right heart failure.

**Table 8:** Clinical phenotype, CKD staging (KDIGO), functional status (NYHA, clinical frailty scale), urinary albumin-to-creatinine ratio category, and pre-admission/discharge pharmacological therapy stratified by precipitating factor group.

Parameter	Behavioral (*n* = 148)	Non-behavioral (*n* = 230)	Unknown (*n* = 108)	*P*-value
Clinical phenotype:				
ADHF	122 (82.4%)	142 (61.7%)	97 (89.8%)	<0.001
Acute pulmonary edema	25 (16.9%)	82 (35.7%)	10 (9.3%)	<0.001
Isolated right HF	1 (0.7%)	1 (0.4%)	0 (0%)	0.704
Cardiogenic shock	0 (0.0%)	5 (2.2%)	1 (0.9%)	0.165
CKD stage (KDIGO):				<0.001
1	18 (12.2%)	22 (9.6%)	4 (3.7%)	
2	57 (38.5%)	95 (41.3%)	29 (26.9%)	
3A	38 (25.7%)	61 (26.5%)	25 (23.1%)	
3B	22 (14.9%)	41 (17.8%)	38 (35.2%)	
4	13 (8.8%)	11 (4.8%)	12 (11.1%)	
Baseline NYHA:				<0.001
I	3 (2%)	16 (7.0%)	0 (0.0%)	
II	37 (25%)	41 (17.8%)	13 (12.0%)	
III	88 (59.5%)	145 (63.0%)	63 (58.3%)	
IV	20 (13.5%)	28 (12.2%)	32 (29.6%)	
Baseline CFS:				0.001
CFS 1–3	13 (8.8%)	23 (10.0%)	4 (3.7%)	
CFS 4–5	86 (58.1%)	138 (60.0%)	46 (42.6%)	
CFS 6–9	49 (33.1%)	69 (30.0%)	58 (53.7%)	
Baseline CFS (Binary):				0.012
CFS ≤4	54 (36.5%)	84 (36.5%)	23 (21.3%)	
CFS ≥5	94 (63.5%)	146 (63.5%)	85 (78.7%)	
UACR category (*n* = 270):				0.796
<30 mg/g Cr	23 (27.7%)	26 (23.2%)	18 (24.0%)	
30–299 mg/g Cr	39 (47.0%)	53 (47.3%)	40 (53.3%)	
>300 mg/g Cr	21 (25.3%)	33 (29.5%)	17 (22.7%)	
Chronic HF medication:				
ARNI	11 (7.4%)	5 (2.2%)	4 (3.7%)	0.041
B-blocker	80 (54.1%)	133 (57.8%)	68 (63.0%)	0.362
MRA	29 (19.6%)	45 (19.6%)	33 (30.6%)	0.052
SGLT2i	27 (18.2%)	40 (17.4%)	31 (28.7%)	0.042
Furosemide	80 (54.1%)	99 (43.0%)	72 (66.7%)	<0.001
ACEi/ARBs	68 (45.9%)	128 (55.7%)	43 (39.8%)	0.016
Discharge HF medication (*n* = 460; 146/215/99):				
ARNIs	16 (11.0%)	27 (12.6%)	7 (7.1%)	0.348
B-blockers	108 (74.0%)	174 (80.9%)	78 (78.8%)	0.287
MRAs	89 (61.0%)	128 (59.5%)	55 (55.6%)	0.691
ACEi/ARBs	57 (39.0%)	95 (44.2%)	21 (21.2%)	<0.001
SGLT2i	98 (67.1%)	133 (61.9%)	63 (63.6%)	0.592
Furosemide	132 (90.4%)	191 (88.8%)	87 (87.9%)	0.808

Values are presented as *n* (%). Between-group comparisons were performed using the chi-square test. *P* values are reported per phenotype category and per individual agent. Discharge medication was recorded in survivors of the index hospitalization (*n* = 460; behavioral *n* = 146, non-behavioral *n* = 215, unknown *n* = 99). ADHF, acute decompensated heart failure; HF, heart failure; KDIGO, Kidney Disease: Improving Global Outcomes; NYHA, New York Heart Association; CFS, clinical frailty scale; UACR, urinary albumin-to-creatinine ratio; ARNI, angiotensin receptor-neprilysin inhibitor; MRA, mineralocorticoid receptor antagonist; SGLT2i, sodium-glucose cotransporter-2 inhibitor; ACEi, angiotensin-converting enzyme inhibitor; ARB, angiotensin receptor blocker.

Pre-admission HF therapy in the unknown group was notable for higher furosemide use (66.7% vs. 54.1% and 43.0%; *p* < 0.001), higher SGLT2i use (28.7% vs. 18.2% and 17.4%; *p* = 0.042), and lower ACEi/ARB use (39.8% vs. 45.9% and 55.7%; *p* = 0.016). At discharge, ACEi/ARB prescription was markedly lower in the unknown group (21.2% vs. 39.0% and 44.2%; *p* < 0.001), almost certainly reflecting the degree of renal impairment constraining renin–angiotensin–aldosterone system (RAAS) optimization. All other discharge medications did not differ significantly between groups (Table 8).

### 3.3 Laboratory and hemodynamic profile

The unknown group exhibited a distinctly more impaired cardio-renal profile (Table 9). Admission eGFR was significantly lower (44 vs. 60.5 and 58 mL/min/1.73m^2^; *p* < 0.001), with both baseline and peak creatinine markedly elevated (baseline: 1.3 vs. 1.1 mg/dl in both; peak: 1.8 vs. 1.4 mg/dl; *p* < 0.001). Urea was substantially higher (85 vs. 52 and 53 mg/dl; *p* < 0.001), and the minimum eGFR during hospitalization was considerably lower (33 vs. 46 and 44 mL/min/1.73m^2^; *p* < 0.001).

**Table 9:** Admission laboratory values and echocardiographic quantitative parameters stratified by precipitating factor group.

Parameter	Behavioral (*n* = 148)	Non-behavioral (*n* = 230)	Unknown (*n* = 108)	*P*-value
Systolic BP (mmHg)	150 [130–170]	155 [130–180]	130 [117–145]	<0.001
eGFR (mL/min/1.73 m^2^)	60.5 [42.8–79.5]	58 [45–77]	44 [29–64.2]	<0.001
Cr (mg/dl)	1.1 [0.9–1.4]	1.1 [0.9–1.4]	1.5 [1.1–1.8]	<0.001
Baseline Cr (mg/dl)	1.1 [0.9–1.3]	1.1 [0.9–1.4]	1.3 [1.0–1.7]	<0.001
Baseline eGFR (mL/min/1.73 m^2^)	62 [45.8–77]	62.5 [47–78]	48 [36–65.2]	<0.001
Maximum Cr (mg/dl)	1.4 [1.1–1.9]	1.4 [1.1–1.9]	1.8 [1.4–2.3]	<0.001
Minimum eGFR (mL/min/1.73 m^2^)	46 [31.5–63]	44 [31–62]	33 [23–48]	<0.001
Urea (mg/dl)	52 [39–73]	53 [42–70]	85 [54.5–111]	<0.001
Albumin (g/dl)	4 [3.8–4.3]	4.1 [3.8–4.3]	4 [3.8–4.2]	0.406
WBC (×10⁹/L)	8.4 [6.6–10.6]	9.8 [7.3–11.8]	7.5 [6.0–8.9]	<0.001
CRP (mg/dl)	0.9 [0.4–2]	1.2 [0.4–2.6]	0.6 [0.4–1.4]	0.001
SGOT (IU/L)	26 [21–35.2]	29 [22–43.8]	26 [20–36]	0.034
SGPT (IU/L)	18 [13–30.2]	21 [14–35]	16 [12–25.2]	0.009
RDW (%)	16.1 [14.9–18.2]	16 [14.6–17.6]	17.1 [15.6–19.6]	<0.001
NT-proBNP (pg/mL)	4681 [2800–10250]	4793 [3078–9310]	8075 [4051–14976]	0.001
Hemoglobin (g/dl)	11.6 [10.5–13]	11.9 [10.5–13]	11.1 [10.1–12.7]	0.104
Hs-Troponin-I (pg/mL)	25.9 [13.9–56.1]	30.3 [12.4–85.6]	31.6 [15.5–60.1]	0.284
Na (mEq/L)	139 [137–141]	139 [137–141]	139 [135–140.2]	0.100
K (mEq/L)	4.5 [4.2–4.9]	4.5 [4.1–4.9]	4.6 [4.2–4.9]	0.260
Cl (mEq/L) [*n* = 353]^a^	103 [100–106]	102 [99.9–105.8]	102 [99.0–105]	0.201
UACR (mg/g Cr) [*n* = 270]^b^	108.7 [27.5–297.2]	115.6 [34.8–382.63]	88 [30.5–275.8]	0.882
Echocardiographic values:				
PASP (mmHg)	45 [35–55]	40 [30–48.8]	50 [40–65]	<0.001
E/e′	16.5 [13.2–22]	16 [13–20.2]	20 [15–26]	<0.001
TV S’ (cm/s)	10.5 [9–12.6]	10.5 [9–12]	9 [8–11]	<0.001
LAVi (mL/m^2^)	51.5 [42.8–63]	50 [42–60]	55 [46–70]	0.003
LVEF (%)	47.5 [30–55]	47.5 [30–55]	45 [25–55]	0.291
TAPSE (mm)	18 [16–20]	18 [16–20]	16.0 [13.5–18]	<0.001
Hospital days (*n* = 460)^c^	7 [5–9]	7 [5–9]	7 [5–9.5]	0.626

Values are presented as median [25th–75th percentile]. Between-group comparisons were performed using the Kruskal–Wallis test. BP, blood pressure; eGFR, estimated glomerular filtration rate (CKD-EPI formula); Cr, creatinine; WBC, white blood cell count; CRP, C-reactive protein; RDW, red cell distribution width; NT-proBNP, N-terminal pro-B-type natriuretic peptide; hs-cTnI, high-sensitivity cardiac troponin I; SGOT, serum glutamic-oxaloacetic transaminase; SGPT, serum glutamic-pyruvic transaminase; Na, sodium; K, potassium; Cl, chloride; UACR, urinary albumin-to-creatinine ratio; PASP, pulmonary artery systolic pressure; E/e′, ratio of mitral inflow early diastolic velocity to mitral annular early diastolic velocity; TV S′, tricuspid lateral annular systolic velocity; LAVi, left atrial volume index indexed to body surface area; LVEF, left ventricular ejection fraction; TAPSE, tricuspid annular plane systolic excursion.

a Chloride measured in *n* = 353 patients.

b UACR measured in *n* = 270 patients.

c Hospital length of stay recorded in survivors (*n* = 460).

NT-proBNP was substantially higher in the unknown group (8075 vs. 4681 and 4793 pg/mL; *p* = 0.001). Admission systolic blood pressure was significantly lower (130 vs. 150 and 155 mmHg; *p* < 0.001). Inflammatory markers were lower in the unknown group (WBC 7.5 vs. 8.4 and 9.8 × 10⁹/L, *p* < 0.001; CRP 0.6 vs. 0.9 and 1.2 mg/dl, *p* = 0.001), while RDW was higher (17.1% vs. 16.1% and 16.0%; *p* < 0.001). Albumin, hemoglobin, troponin, electrolytes, and UACR did not differ significantly between groups (Table 9).

### 3.4 Echocardiographic characteristics

Despite a comparable LVEF distribution across groups (*p* = 0.609), the echocardiographic profile of the unknown group was markedly distinct in terms of RV and valvular pathology. RV dilation was three times as prevalent in the unknown group as in the non-behavioral/clinical group (44.4% vs. 27.0% behavioral and 14.3% non-behavioral/clinical; *p* < 0.001). Severe tricuspid regurgitation was present in 39.8% versus 16.9% and 12.6% (*p* < 0.001), and moderate-to-severe mitral regurgitation was more prevalent (55.6% vs. 37.2% and 41.3%; *p* = 0.006). The echocardiographic probability of PH was high in 68.5% of unknown patients versus 58.1% behavioral and 41.3% non-behavioral (*p* < 0.001) (Table 10).

**Table 10:** Echocardiographic characteristics stratified by precipitating factor group.

Echocardiographic parameter	Behavioral (*n* = 148)	Non-behavioral (*n* = 230)	Unknown (*n* = 108)	*P*-value
LV dilation	42 (28.4%)	49 (21.3%)	31 (28.7%)	0.187
RV dilation	40 (27.0%)	33 (14.3%)	48 (44.4%)	<0.001
LVEF:				0.609
≥50%	74 (50%)	115 (50%)	50 (46.3%)	
41%–49%	14 (9.5%)	21 (9.1%)	6 (5.6%)	
≤40%	60 (40.5%)	94 (40.9%)	52 (48.1%)	
Tricuspid regurgitation:				<0.001
No/Mild	86 (58.1%)	147 (63.9%)	38 (35.2%)	
Moderate	37 (25.0%)	54 (23.5%)	27 (25.0%)	
Severe	25 (16.9%)	29 (12.6%)	43 (39.8%)	
Mitral regurgitation:				0.006
No/Mild	93 (62.8%)	135 (58.7%)	48 (44.4%)	
Moderate	42 (28.4%)	79 (34.3%)	41 (38.0%)	
Severe	13 (8.8%)	16 (7.0%)	19 (17.6%)	
Mitral stenosis:				0.543
No/Mild	143 (96.6%)	219 (95.2%)	102 (94.4%)	
Moderate	3 (2.0%)	10 (4.3%)	4 (3.7%)	
Severe	2 (1.4%)	1 (0.4%)	2 (1.9%)	
Aortic stenosis:				0.147
No/Mild	124 (83.8%)	179 (77.8%)	82 (75.9%)	
Moderate	11 (7.4%)	22 (9.6%)	6 (5.6%)	
Severe	13 (8.8%)	29 (12.6%)	20 (18.5%)	
Aortic regurgitation:				0.354
No/Mild	134 (90.5%)	199 (86.5%)	91 (84.3%)	
Moderate	13 (8.8%)	29 (12.6%)	14 (13.0%)	
Severe	1 (0.7%)	2 (0.9%)	3 (2.8%)	
Probability of PH:				<0.001
Low	19 (12.8%)	43 (18.7%)	5 (4.6%)	
Moderate	43 (29.1%)	92 (40.0%)	29 (26.9%)	
High	86 (58.1%)	95 (41.3%)	74 (68.5%)	

Values are presented as *n* (%). Between-group comparisons were performed using the chi-square test. LVEF = left ventricular ejection fraction measured by Simpson's biplane method, categorized as preserved (≥50%), mildly reduced (41%–49%), or reduced (≤40%); LV = left ventricle; RV = right ventricle; PH = pulmonary hypertension. Valvular disease severity was graded as no/mild, moderate, or severe according to current echocardiographic guidelines. PH probability was classified as low, moderate, or high based on estimated pulmonary artery systolic pressure and additional indirect echocardiographic signs as described in Section 2.3 and Supplementary Table S1.

Quantitative RV functional parameters confirmed these findings: TAPSE was lower (16.0 vs. 18 mm; *p* < 0.001), TV S' was reduced (9 vs. 10.5 cm/s in both groups; *p* < 0.001), and PASP was elevated (50 vs. 45 and 40 mmHg; *p* < 0.001). The E/e′ ratio was higher (20 vs. 16.5 and 16; *p* < 0.001) and LAVi was larger (55 vs. 51.5 and 50 mL/m^2^; *p* = 0.003) (Table 9).

### 3.5 In-hospital outcomes

In-hospital outcomes were significantly worse in the unknown group. All-cause in-hospital mortality was 8.3% compared to 1.4% (behavioral) and 6.5% (non-behavioral; *p* = 0.027). Cardiovascular death occurred in 6.5% versus 0% and 5.2% (*p* = 0.011). Need for in-hospital RRT was 4.6% versus 0% and 3.0% (*p* = 0.046). Hospital length of stay was similar across groups (7 days; *p* = 0.626). Over the 12 months of follow-up, 11 patients (2.3%) progressed to terminal RRT — one in the behavioral, five in the non-behavioral/clinical and five in the unknown group — with cumulative incidences of 1.6% by 3 months and 2.1% by 6 months (Tables 9, 11).

**Table 11:** In-hospital outcomes, primary and secondary endpoints, and follow-up events stratified by precipitating factor group.

Endpoints	Behavioral (*n* = 148)	Non-behavioral (*n* = 230)	Unknown (*n* = 108)	*P*-value
Triple endpoint (6M)	45 (30.4%)	80 (34.8%)	54 (50.0%)	0.004
Triple endpoint (12M)	61 (41.2%)	109 (47.4%)	70 (64.8%)	0.001
In hospital:				
RRT	0 (0.0%)	7 (3.0%)	5 (4.6%)	0.046
All-cause death	2 (1.4%)	15 (6.5%)	9 (8.3%)	0.027
Cardiovascular death	0 (0.0%)	12 (5.2%)	7 (6.5%)	0.011
Primary endpoint:				
Death (3M)	11 (7.4%)	25 (10.9%)	22 (20.4%)	0.005
Death (6M)	19 (12.8%)	39 (17.0%)	31 (28.7%)	0.004
Death (12M)	30 (20.3%)	68 (29.6%)	45 (41.7%)	0.001
Secondary endpoint:				
Terminal RRT by 3 months	0 (0.0%)	4 (1.7%)	4 (3.7%)	0.070
Terminal RRT by 6 months	1 (0.7%)	5 (2.2%)	4 (3.7%)	0.238
Terminal RRT by 12 months	1 (0.7%)	5 (2.2%)	5 (4.6%)	0.109
Rest endpoints:				
3 months (*n* = 421; 137/201/83):				
ED visit (any cause)	64 (46.7%)	74 (36.8%)	38 (45.8%)	0.138
Rehospitalization (any cause)	40 (29.2%)	52 (25.9%)	26 (31.3%)	0.605
Rehospitalization (for AHF)	18 (13.1%)	28 (13.9%)	14 (16.9%)	0.733
6 months (*n* = 386; 128/186/72):				
ED visit (any cause)	75 (58.6%)	91 (48.9%)	40 (55.6%)	0.221
Rehospitalization (any cause)	50 (39.1%)	67 (36.0%)	26 (36.1%)	0.846
Rehospitalization (for AHF)	25 (19.5%)	36 (19.4%)	18 (25.0%)	0.571
12 months (*n* = 335; 118/158/59):				
ED visit (any cause)	78 (66.1%)	96 (60.8%)	41 (69.5%)	0.424
Rehospitalization (any cause)	57 (48.3%)	65 (41.1%)	33 (55.9%)	0.130
Rehospitalization (for AHF)	31 (26.3%)	37 (23.4%)	22 (37.3%)	0.120

Values are presented as *n* (%). Between-group comparisons were performed using the chi-square test. RRT, renal replacement therapy; AHF, acute heart failure; ED, emergency department. All-cause death was the primary endpoint of the study. Need for RRT constituted the secondary endpoint. Follow-up events (ED visits and rehospitalizations) were recorded only in patients who survived the index hospitalization and did not require RRT; the denominator decreases over time due to attrition from death and RRT initiation. The denominator at each time point is the number of patients with a recorded assessment and differs slightly from the number free of death and terminal RRT at that time: four assessments were not recorded (three at 6 months and one at 12 months) and two were recorded in the interval in which the patient reached an endpoint (one at 3 months and one at 6 months) (3 months: *n* = 421; 6 months: *n* = 386; 12 months: *n* = 335). Group-specific denominators for the follow-up events are given in the corresponding row headings as behavioral/non-behavioral/unknown. The triple composite endpoint comprises all-cause death, AHF rehospitalization, or renal replacement therapy. Because ED visits and rehospitalizations were ascertained only in patients who remained alive and free of terminal RRT, the proportions shown for these endpoints are conditional on survival and are not first-event rates; the competing-risk analysis of first AHF rehospitalization is reported in Section 3.7 and Supplementary Table S7.

### 3.6 Mid-term outcomes and survival analysis

Mortality in the unknown group rose steeply at all timepoints: 20.4% at 3 months (vs. 7.4% behavioral and 10.9% non-behavioral; *p* = 0.005), 28.7% at 6 months (vs. 12.8% and 17.0%; *p* = 0.004), and 41.7% at 12 months (vs. 20.3% and 29.6%; *p* = 0.001). The triple composite endpoint was reached by 50.0% of the unknown group at 6 months and 64.8% at 12 months, compared to 30.4–34.8% and 41.2–47.4% respectively in the other groups (*p* = 0.004 and *p* = 0.001). ED visits and AHF-specific rehospitalization rates, assessed among patients remaining alive and free of terminal RRT, did not differ significantly between groups at any timepoint (Table 11). These proportions are conditional on survival and on freedom from terminal RRT, and their denominators decline fastest in the group with the highest mortality; they are therefore not first-event rates and should be read together with the competing-risk analysis presented in Section 3.7.

Kaplan–Meier analysis confirmed these findings. For the primary endpoint of all-cause death, the overall log-rank p was <0.001, with survival at 12 months of 79.7% (95% CI 72.3–85.4%) in the behavioral, 70.4% (64.1–75.9%) in the non-behavioral/clinical and 58.3% (48.5–67.0%) in the unknown group; all three pairwise comparisons were significant (behavioral vs. non-behavioral *p* = 0.044; behavioral vs. unknown *p* < 0.001; non-behavioral vs. unknown *p* = 0.018). A parallel gradient was observed for the death/RRT composite (overall log-rank *p* < 0.001). For the triple composite, the unknown group again showed significantly inferior event-free survival to both the behavioral (pairwise *p* < 0.001) and the non-behavioral group (pairwise *p* = 0.003), while the latter two did not differ from each other (*p* = 0.189); event-free survival at 12 months was 35.2% (26.3–44.2%) in the unknown group, 52.6% (46.0–58.8%) in the non-behavioral and 58.8% (50.4–66.2%) in the behavioral group (Figures 1–3).

**Figure 1:**
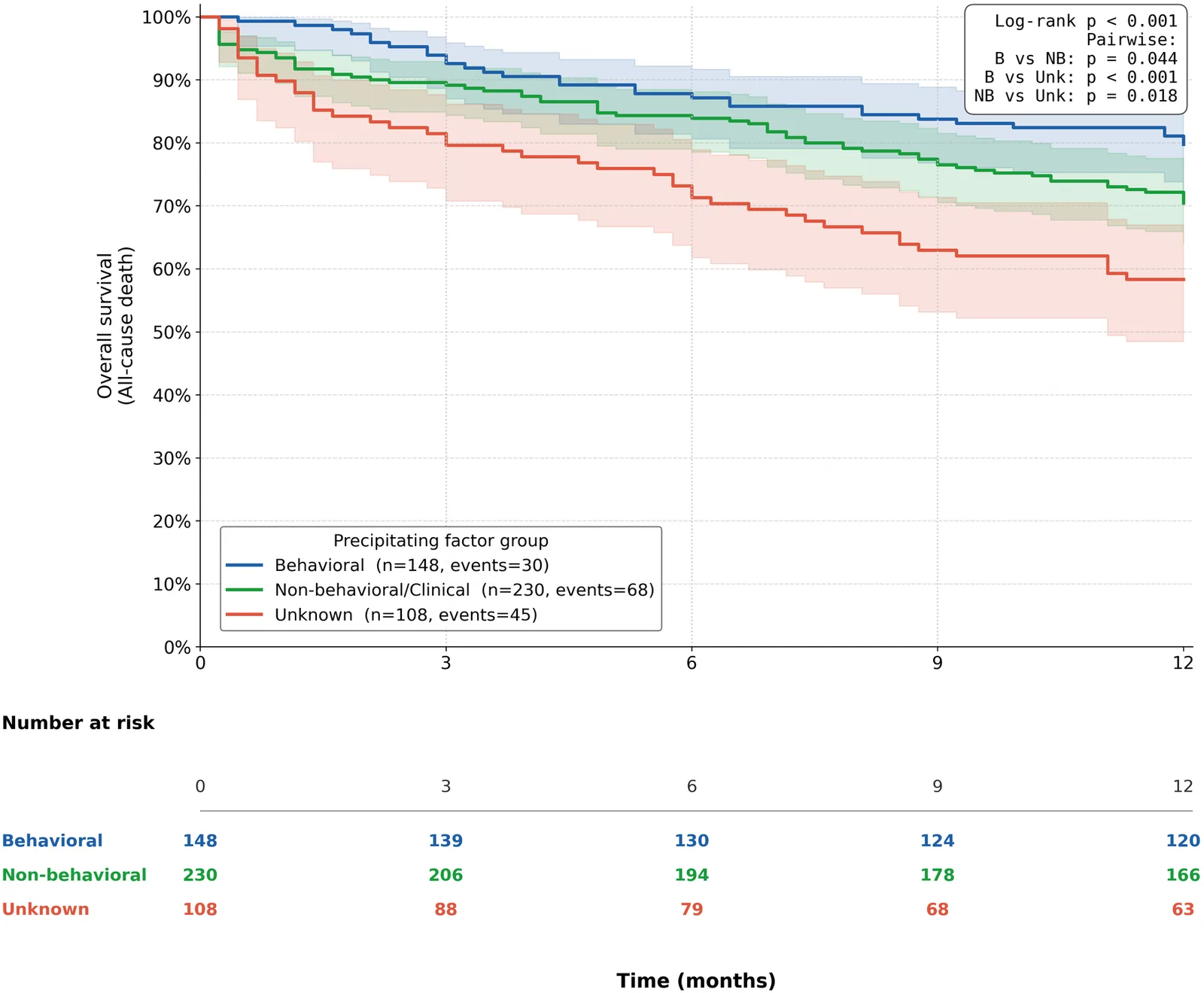
Kaplan–Meier overall survival curves for all-cause mortality (primary endpoint) stratified by precipitating factor group. Survival at 12 months was 79.7% in the behavioral, 70.4% in the non-behavioral/clinical and 58.3% in the unknown group. Overall log-rank *p* < 0.001; pairwise behavioral vs. non-behavioral *p* = 0.044, behavioral vs. unknown *p* < 0.001, non-behavioral vs. unknown *p* = 0.018. Shaded areas represent 95% confidence intervals; numbers at risk are shown below the *x*-axis.

**Figure 2:**
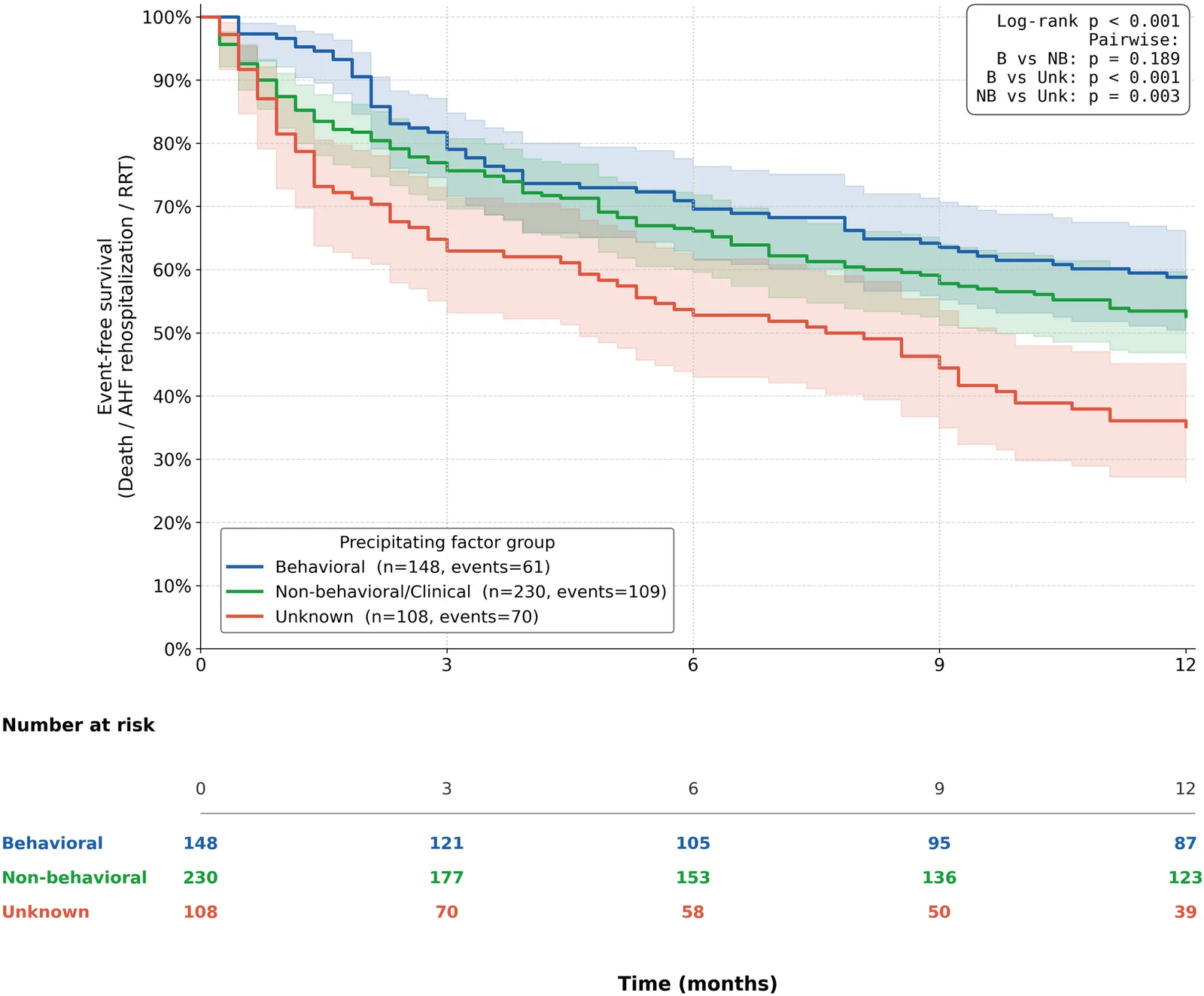
Kaplan–Meier event-free survival curves for the triple composite endpoint (all-cause death, AHF rehospitalization or renal replacement therapy) stratified by precipitating factor group. Event-free survival at 12 months was 58.8% in the behavioral, 52.6% in the non-behavioral/clinical and 35.2% in the unknown group. Overall log-rank *p* < 0.001; pairwise behavioral vs. non-behavioral *p* = 0.189, behavioral vs. unknown *p* < 0.001, non-behavioral vs. unknown *p* = 0.003. Shaded areas represent 95% confidence intervals; numbers at risk are shown below the *x*-axis.

**Figure 3:**
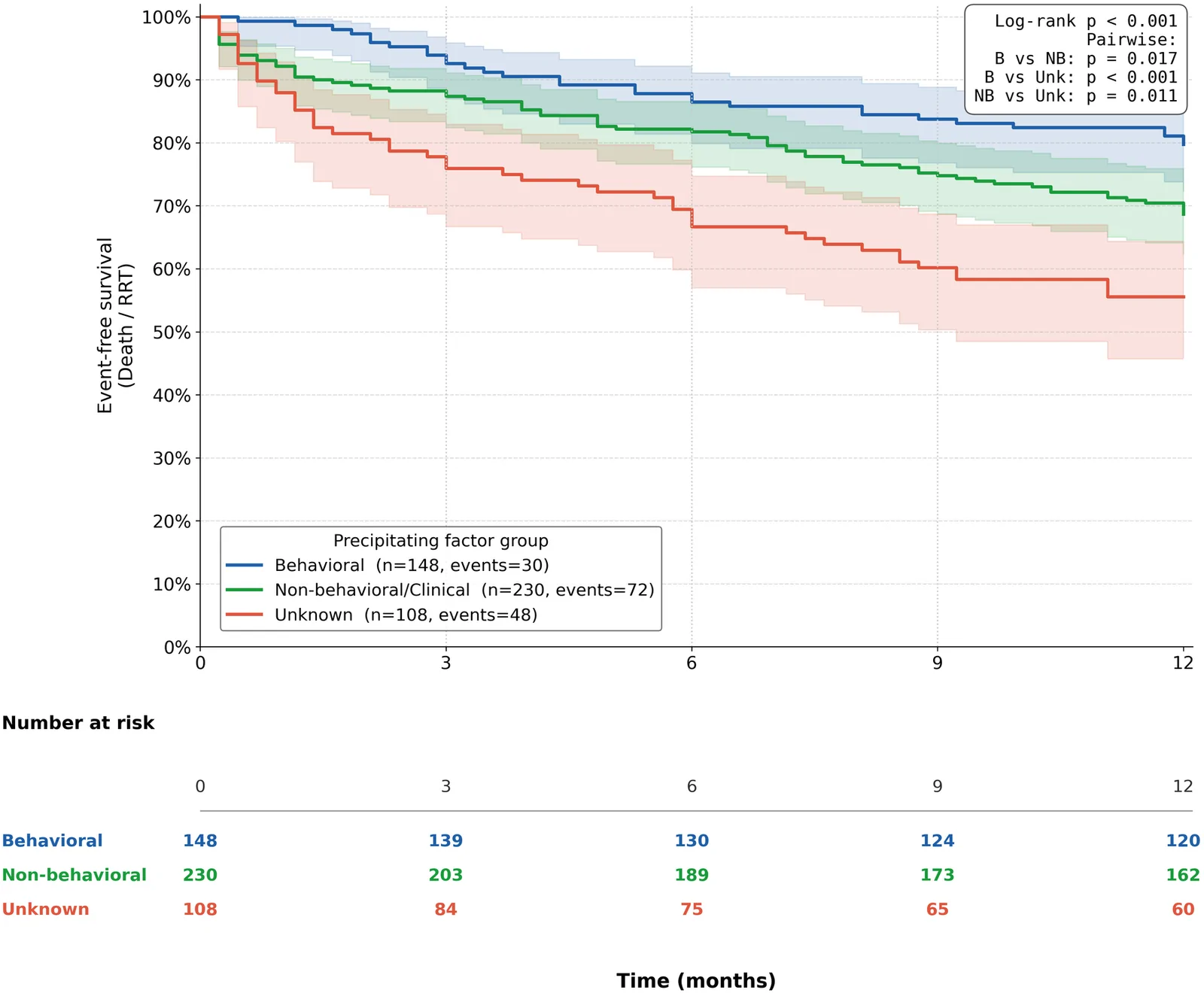
Kaplan–Meier event-free survival curves for the composite endpoint of all-cause death or terminal renal replacement therapy, stratified by precipitating factor group. Event-free survival at 12 months was 79.7% in the behavioral, 68.7% in the non-behavioral/clinical and 55.6% in the unknown group. Overall log-rank *p* < 0.001; pairwise behavioral vs. non-behavioral *p* = 0.017, behavioral vs. unknown *p* < 0.001, non-behavioral vs. unknown *p* = 0.011. Shaded areas represent 95% confidence intervals; numbers at risk are shown below the *x*-axis.

Unadjusted Cox proportional hazards regression quantified these between-group differences. With the behavioral group as the reference, the unknown group had a markedly elevated hazard for all-cause death (HR 2.44, 95% CI 1.54–3.88; *p* < 0.001), for the death/RRT composite (HR 2.69, 95% CI 1.70–4.24; *p* < 0.001) and for the triple composite endpoint (HR 1.96, 95% CI 1.39–2.77; *p* < 0.001). The non-behavioral/clinical group showed significantly elevated hazards for mortality (HR 1.55, 95% CI 1.01–2.38; *p* = 0.045) and the death/RRT composite (HR 1.67, 95% CI 1.09–2.56; *p* = 0.018) but not for the triple composite (HR 1.24, 95% CI 0.90–1.69; *p* = 0.186). In a complementary contrast with the non-behavioral group as the reference, the unknown group remained at substantially higher risk across all three endpoints (all-cause mortality: HR 1.58, 95% CI 1.08–2.30, *p* = 0.018; death/RRT: HR 1.61, 95% CI 1.12–2.32, *p* = 0.011; triple composite: HR 1.59, 95% CI 1.18–2.15, *p* = 0.003). The proportional hazards assumption was satisfied in all of these unadjusted models.

In a pre-specified sensitivity analysis excluding the 26 patients who died during the index hospitalization (*n* = 460), the prognostic separation between groups was maintained (overall log-rank *p* = 0.002). Event-free survival at 12 months was 59.6% (95% CI 51.2–67.0%) in the behavioral group, 56.3% (49.4–62.6%) in the non-behavioral group, and 38.4% (28.9–47.8%) in the unknown group (pairwise: behavioral vs. unknown *p* < 0.001; non-behavioral vs. unknown *p* = 0.003; behavioral vs. non-behavioral *p* = 0.547), indicating that the prognostic divergence of the unknown group persisted after discharge and was not explained solely by in-hospital mortality (Supplementary Figure S1).

### 3.7 Competing-risk analysis of AHF rehospitalization

Because non-fatal events could only be observed in patients who remained alive and free of terminal RRT, and because attrition was fastest in the group with the highest mortality, the cumulative incidence of first AHF rehospitalization was re-estimated with death and terminal RRT as competing events. This approach recovered 53 first AHF rehospitalizations absent from the survivor-conditional follow-up field — 51 in patients who subsequently died and two in patients who subsequently initiated terminal RRT, all of whom had by then left the survivor-based denominator — yielding 143 first rehospitalizations across the cohort. The 12-month cumulative incidence of first AHF rehospitalization was 29.7% (95% CI 22.6–37.2%) in the behavioral, 26.1% (20.6–31.9%) in the non-behavioral/clinical and 36.1% (27.1–45.1%) in the unknown group, with corresponding cumulative incidences of the competing event of 11.5%, 21.3% and 28.7% respectively. The between-group difference in rehospitalization was not statistically significant (Gray's test *p* = 0.207), and the Fine–Gray subdistribution model showed no significant effect of precipitant category [unknown vs. behavioral subdistribution HR (sHR) 1.25, 95% CI 0.82–1.93; non-behavioral vs. behavioral sHR 0.87, 0.59–1.29]. After appropriate accounting for informative censoring, the prognostic divergence between groups therefore remains attributable to mortality rather than to a higher burden of AHF rehospitalization (Supplementary Figure S3, Supplementary Table S7).

### 3.8 Multivariable analysis and incremental prognostic value

To determine whether precipitant category conveys prognostic information beyond the clinical features that define it, multivariable Cox models including age, admission eGFR, RHF, log NT-proBNP and baseline frailty were compared with and without precipitant category (*n* = 475 with complete covariate data). For the primary endpoint of all-cause death, adding precipitant category did not significantly improve model fit (likelihood ratio *χ*^2^ = 5.43, df = 2, *p* = 0.066; C-index 0.716 vs. 0.724), and the unknown group was no longer independently associated with outcome (adjusted HR 1.43, 95% CI 0.88–2.31). The same was true for the triple composite (likelihood ratio *χ*^2^ = 3.60, *p* = 0.165; adjusted HR for the unknown group 1.33, 95% CI 0.93–1.91). For the death/RRT composite, model fit improved modestly (likelihood ratio *χ*^2^ = 8.46, *p* = 0.015), but this improvement was driven by the contrast between the behavioral and non-behavioral/clinical groups (adjusted HR 1.85, 95% CI 1.20–2.85) rather than by the unknown group (adjusted HR 1.55, 95% CI 0.97–2.50). Across all models, RHF, NT-proBNP and frailty remained independently associated with outcome, and admission eGFR in two of the three (*p* = 0.061 for all-cause death), whereas age did not. The proportional hazards assumption was satisfied in all three multivariable models (Supplementary Table S5).

Because renal function at admission reflects both pre-existing CKD and the acute decrement that accompanies decompensation, the contribution of each component was examined separately (Supplementary Tables S8 and S9). Baseline renal impairment was more severe in the unknown group — baseline eGFR 48 [36–65.2] versus 62 [45.8–77] and 62.5 ([47–78] mL/min/1.73 m^2^ (*p* < 0.001), and KDIGO stage 3B–4 in 46.3% versus 23.6% and 22.6% (*p* < 0.001) — whereas the change in eGFR from baseline to admission did not differ significantly between groups (−1 [−9 to 2] versus 0 [−4.2 to 4] and −1 [−6 to 2.8] mL/min/1.73 m^2^; *p* = 0.083). Acute kidney injury during the index hospitalization, which is staged on the peak in-hospital creatinine rather than on the admission value, was nevertheless more frequent in the unknown group (64.8% versus 41.9% and 46.5%; *p* < 0.001), indicating that the additional renal deterioration in this group was captured by the in-hospital peak rather than by the value at presentation. In the whole cohort (*n* = 486), adjustment for admission eGFR attenuated the unadjusted mortality HR of the unknown group from 2.44 to 1.85 (95% CI 1.16–2.96), whereas adjustment for baseline eGFR or for KDIGO stage 3B–4 attenuated it less (2.04, 1.28–3.26 and 2.09, 1.31–3.34 respectively); entering both renal measures together left the estimate unchanged at 1.85 (1.15–2.96), with baseline eGFR no longer independently associated with mortality (*p* = 0.115). In the full multivariable model (*n* = 475) the unknown group remained non-significant whichever renal variable was used (adjusted HR 1.43, 0.88–2.31 with admission eGFR; 1.50, 0.93–2.42 with baseline eGFR; 1.50, 0.92–2.43 with KDIGO stage 3B–4), and the likelihood ratio test for precipitant category was non-significant in each, although borderline for the baseline-eGFR model (*p* = 0.066, 0.050 and 0.055). The association was therefore attenuated more by adjustment for renal function at presentation than by adjustment for the chronic stage, and the conclusions of the study are unchanged by the choice of renal parameterization. The proportional hazards assumption was satisfied for the models using admission eGFR and categorical CKD stage but was violated for baseline eGFR modeled as a continuous variable (Schoenfeld *p* = 0.01); that model is therefore interpreted with caution and the categorical CKD-stage analysis is the preferred alternative parameterization.

### 3.9 Sensitivity analysis excluding *de novo* heart failure

*De novo* HF accounted for 132 of 486 patients (27.2%) and was least frequent in the unknown group (13.9%, vs. 22.3% behavioral and 36.5% non-behavioral/clinical). After excluding these patients, leaving 354 with chronic HF (behavioral *n* = 115, non-behavioral/clinical *n* = 146, unknown *n* = 93), the prognostic separation of the unknown group from the behavioral group was not attenuated but strengthened. For the primary endpoint the overall log-rank p was <0.001, with survival at 12 months of 76.5% in the behavioral, 63.0% in the non-behavioral/clinical and 51.6% in the unknown group, and an unadjusted HR for the unknown group of 2.53 (95% CI 1.57–4.07) versus 2.44 in the full cohort; corresponding HRs were 2.80 (1.75–4.49) for the death/RRT composite and 2.03 (1.42–2.91) for the triple composite. In this restricted cohort the behavioral group also separated significantly from the non-behavioral/clinical group for all three endpoints (mortality HR 1.71, 95% CI 1.08–2.71; death/RRT HR 1.89, 1.20–2.98; triple composite HR 1.52, 1.08–2.13), a contrast that had been weaker in the full cohort. The contrast that weakened in this restricted cohort was non-behavioral/clinical versus unknown, which lost statistical significance for all-cause death (*p* = 0.052, versus *p* = 0.018 in the full cohort) and for the triple composite (*p* = 0.073, versus *p* = 0.003), and was attenuated though still significant for the death/RRT composite (*p* = 0.045, versus *p* = 0.011) (Supplementary Figure S2, Supplementary Table S6).

## 4 Discussion

### 4.1 Principal finding

In this prospective single-center cohort of consecutive AHF admissions, patients in whom no precipitant could be identified formed a clinically recognizable subgroup: older, frailer, more burdened by advanced CKD and multivalvular disease, with predominant RV involvement and markedly worse 12-month survival than patients with behavioral or clinical precipitants. Two further observations qualify this picture. First, the survival disadvantage of this group was accounted for by the clinical features that define it — after adjustment for age, renal function, RHF, natriuretic peptide level and frailty, the designation no longer carried independent prognostic weight. Second, the disadvantage appeared to be driven predominantly by mortality rather than by differences in first AHF rehospitalization, the cumulative incidence of which did not differ significantly between groups once death and terminal RRT were treated as competing events. Taken together, these findings suggest that the absence of an identifiable trigger marks a patient whose decompensation reflects the trajectory of advanced multimorbid disease rather than a discrete precipitating event — an interpretation that our data support but cannot establish.

### 4.2 Population characteristics and comparability with existing registries

The overall profile of our cohort is broadly comparable to contemporary European AHF registries in presentation pattern and etiology, while differing substantially in age. Our median age of 81 years exceeds that reported in most landmark registries, including EHFS II and Gulf CARE (4, 5), consistent with the documented aging of European and other high-income AHF populations (6, 7). Comorbidity burden was correspondingly higher, particularly for arterial hypertension and AF, both of which are strongly age-dependent; residual differences in ascertainment methodology and population selection cannot be excluded, and direct causal attribution to age alone is not possible without individual-level adjustment (6, 8).

The distribution of clinical phenotypes closely mirrored EHFS II, with decompensated HF dominant and APO second (4), reinforcing the consistency of this hierarchy across settings and time periods. *De novo* HF was somewhat less frequent than in EHFS II, plausibly reflecting the higher proportion of longstanding established disease in an older cohort. Valvular etiology was more prevalent than in most published registries — a finding that may reflect local referral patterns, the age of our population, or both, and warrants cautious interpretation.

### 4.3 Precipitating factors: distribution and the unknown phenotype in context

The distribution of precipitants in our cohort largely mirrors that of major AHF registries, with dietary non-compliance, uncontrolled hypertension, arrhythmia and infection dominating, broadly consistent with GREAT, Gulf CARE and EHFS II (4, 5, 9, 10). Multiple concurrent precipitants were common, illustrating the multifactorial nature of decompensation.

The proportion of patients with no identifiable precipitant falls within, though toward the lower end of, the published range — from 14% in the CHARM program (11) to as much as 44% in GREAT (9). This variation most likely reflects differences in the rigor of ascertainment, case mix and clinical definitions rather than true biological differences. In our study the precipitant was documented prospectively by the admitting cardiologist as a mandatory field of the departmental protocol, which may explain the relatively lower proportion of unknown cases compared with registries relying on unstructured investigator judgment.

One apparent inconsistency deserves comment. Chronic AF was considerably more prevalent in the unknown group, yet AF was never assigned as the precipitant in those patients. This is definitional rather than contradictory: arrhythmia was classified as a precipitant only when new in onset or when a rapid ventricular response was judged temporally and hemodynamically responsible for the decompensation. In patients with long-standing permanent AF and adequate rate control, the arrhythmia is a chronic substrate rather than an acute trigger, and its high prevalence in this group is better read as a further marker of advanced atrial and right-sided remodeling.

The prognostic implications of an unknown precipitant appear more adverse in our cohort than in some previous analyses. Platz et al. found no significant mortality difference associated with an unknown precipitant (11), whereas we observed markedly worse 12-month mortality. This discrepancy likely reflects the older age, higher frailty burden and greater CKD prevalence of our cohort relative to the CHARM population, though we cannot exclude that a more structured ascertainment process captured a more severely ill and less recoverable subgroup.

### 4.4 The unknown precipitant as a coherent clinical phenotype

Several converging clinical and echocardiographic features support the internal coherence of this phenotype. Lower admission systolic blood pressure, lower WBC and CRP despite markedly higher NT-proBNP, absence of a hypertensive or infectious trigger, a high prevalence of chronic AF and prior loop diuretic use together point toward a low-output, chronically impaired hemodynamic state with diminished reserve rather than an acute stress superimposed on previously compensated disease. The elevation in NT-proBNP should not, however, be attributed to neurohormonal activation alone: it is partly explained by the higher prevalence of AF, the more advanced renal impairment and the older age of this group, all of which independently raise natriuretic peptide concentrations.

The echocardiographic signature was equally characteristic, with RV dilation, severe tricuspid regurgitation, elevated PASP and reduced TAPSE — consistent with established RV failure and PH, a phenotype known to carry poor prognosis and to be substantially refractory to standard HF therapies (12, 13). Notably, the distribution of LVEF did not differ across groups, indicating that the excess risk is not explained by LV systolic dysfunction alone but by the additional burden of right heart involvement and cardio-renal impairment (12, 13). The prognostic separation was preserved in a sensitivity analysis excluding patients who died during the index hospitalization, indicating that the separation persisted after discharge and was not explained solely by in-hospital mortality (Supplementary Figure S1).

### 4.5 What the unknown-precipitant designation does and does not add

Three analyses delineate the limits of what can be inferred from this categorization, and we present them together because each constrains the interpretation of the others.

First, once age, renal function, RHF, natriuretic peptide level and frailty were entered into a multivariable model, precipitant category no longer improved model fit for the primary endpoint, and the unknown group lost independent significance for every endpoint examined. We interpret this not as a null result but as consistent with the premise of the study: the excess risk of this group is largely accounted for by the advanced cardiorenal, right-sided and frailty burden that the designation aggregates rather than by the absence of a trigger as such. The same conclusion survives the alternative renal parameterizations examined in Section 3.8: substituting baseline eGFR or CKD stage for admission eGFR left both the adjusted HR and the likelihood ratio test essentially unchanged, indicating that the attenuation reflects the cardiorenal severity of the phenotype at presentation rather than an artifact of how renal function was modeled. The label is therefore best understood as an integrative bedside descriptor rather than an independent risk factor in the conventional regression sense. Its only potential advantage is immediacy — it is available on the day of admission, before natriuretic peptide results, formal frailty scoring or RV assessment have been assimilated into a clinical judgment. Whether acting on this signal improves outcomes is a hypothesis these data generate but cannot test.

Second, the competing-risk analysis converts what would otherwise be a vulnerable claim into a defensible one. In a survivor-conditional analysis, the observation that AHF rehospitalization rates did not differ between groups would be uninterpretable, because the group with the highest mortality contributes the fewest person-months at risk and its patients are removed from observation before they can be rehospitalized. Re-estimating cumulative incidence with death and terminal RRT as competing events recovered 53 rehospitalizations that the conditional approach had discarded, the large majority of them in patients who subsequently died, yet the between-group difference remained non-significant. The excess risk of this phenotype therefore appears to be carried predominantly by mortality rather than by a higher burden of first AHF rehospitalization — a distinction with direct implications for which interventions could plausibly modify it.

Third, it has been suggested that patients presenting with *de novo* HF might inflate the apparent risk of the unknown group, since their etiology may later be identified and reclassified. In our cohort *de novo* presentations were least frequent in the unknown group, and excluding them strengthened rather than attenuated the separation of the unknown group from the behavioral group, although the contrast between the unknown and the non-behavioral/clinical groups weakened (Section 3.9). The finding is therefore not an artifact of incomplete diagnostic workup in newly presenting patients.

A complementary observation emerges from the same models. After adjustment, patients with a behavioral precipitant retained a significantly lower hazard of death and of death or RRT than those with clinical precipitants (Supplementary Table S5). A comparable unadjusted contrast was present in the cohort restricted to chronic HF (Supplementary Table S6), although the two estimates are not directly comparable because the latter is unadjusted. Because behavioral precipitants are by definition modifiable, this may be the more directly actionable of the two findings reported here.

### 4.6 Guideline-directed medical therapy: underuse and therapeutic inertia

The significantly lower rate of RAAS inhibitor prescription at discharge in the unknown group merits particular attention, given that these agents are disease-modifying and confer both mortality and cardiorenal benefit (1, 14, 15). The pattern likely reflects a combination of reduced drug tolerance driven by advanced renal impairment and, potentially, therapeutic inertia in older and more complex patients. The second mechanism cannot be proven from observational data, and for some patients lower prescription may be clinically appropriate. Nonetheless, the pattern is consistent with the QUALIFY registry, in which guideline adherence was lowest in precisely the groups with the highest residual risk (16), and with CHAMP-HF, where renal impairment and advanced age were principal barriers to complete guideline-directed medical therapy (GDMT) (17).

Randomized data specifically addressing the very elderly, frail and renally impaired remain sparse, and perceived risks of electrolyte disturbance, hypotension and worsening renal function may outweigh perceived benefit in practice. Subgroup analyses of landmark trials nonetheless demonstrate preserved relative risk reduction across strata of age, renal function and comorbidity, suggesting that the benefit-to-risk balance remains favorable when therapy is initiated cautiously and titrated with close monitoring (18–20).

Beyond titration itself, the model of care matters. Follow-up by specialist HF nurses has been associated with greater achievement of target GDMT doses and with lower mortality, and represents a plausible delivery mechanism for the intensified surveillance that this phenotype would require (21).

### 4.7 From behavioral to unknown: a continuum of risk

A graded rather than dichotomous risk pattern emerges when endpoints are examined separately: patients with a behavioral precipitant carry the lowest hazard, those with clinical precipitants an intermediate hazard, and those without an identifiable precipitant the highest. This gradient supports the interpretation that precipitant category reflects a continuum of underlying disease severity and reversibility rather than a sharp division between identifiable and non-identifiable triggers. The intermediate position of the non-behavioral group is consistent with registry data showing that specific clinical precipitants exert markedly different prognostic effects. In the intercontinental GREAT registry, acute coronary syndrome and infection independently increased 90-day mortality while AF was associated with lower mortality (9); an analysis of the Get With The Guidelines–Heart Failure registry similarly showed that myocardial ischemia, worsening renal function and lower respiratory tract infection raise short-term mortality, whereas dietary non-compliance is associated with lower post-discharge death across ejection fraction strata (22). Because our non-behavioral/clinical group encompasses both higher-risk triggers and more reversible ones, its intermediate hazard plausibly reflects this internal heterogeneity (23–25).

### 4.8 Non-adherence and the behavioral precipitant: a preventable burden

Nearly one in three patients had dietary or pharmacological non-compliance as the predominant precipitant, consistent with international estimates that medication non-adherence affects 30%–50% of HF patients and substantially raises the risk of hospitalization and death (26, 27). Whether this reflects a genuinely higher prevalence of non-adherence in our population, or a lower threshold for attributing decompensation to behavioral factors when no clear clinical trigger is present, cannot be determined from our data; residual misclassification is possible and is compounded by our convention of assigning ambiguous mixed cases to the behavioral group.

Non-adherence is multifactorial. Our patients are elderly and frequently frail, with objective limitations in managing complex regimens and dietary restriction; at the same time, the workload of acute care limits the time available for education and reinforcement at discharge. Structured written discharge instructions, scheduled early follow-up, multidisciplinary nurse-led programs and home-based care models have all shown benefit in this context (21, 28). Digital health tools may in future free clinical time for the patient–physician interaction where the most impactful preventive conversations occur, though evidence in elderly AHF populations remains limited (29, 30).

Nearly half of the cohort reached the composite endpoint within 12 months, ranging from 41.2% in the behavioral to 64.8% in the unknown group — concordant with contemporary European registry data and a reminder that AHF hospitalization is a sentinel event requiring intensive post-discharge care whatever the precipitant (31).

## 5 Limitations

Several limitations qualify these findings. As a single-center study, generalizability is limited. Recruitment was confined to the Cardiology Department, and patients with AHF admitted under Internal Medicine — typically older and more comorbid — were not captured, which may have led to underestimation of the frailest end of the AHF spectrum. Our cohort is also considerably older than most published registries, raising the possibility that patients in prior cohorts had more reversible precipitants and greater hemodynamic reserve; cross-study comparisons should be read with this in mind.

The identification of a precipitant is, moreover, a cross-sectional snapshot of a single admission. The same patient may present with an identifiable trigger at one hospitalization and without one at the next, implying a degree of inherent variability in group assignment that no observational design can control. Group membership should therefore not be treated as a fixed patient characteristic, and precipitant category should not be used as a standalone prognostic label in individual patients even though it carries clear signal at the population level.

Precipitant documentation was performed prospectively by the admitting physician as part of routine care, and the documented precipitant was mapped to one of the three categories by two investigators working by consensus rather than through duplicate independent adjudication. No record of discordant independent assignments was retained, and a Cohen's kappa cannot therefore be reconstructed *post hoc*; reproducibility of the categorization across centers remains to be established. The investigations available to that judgment were uniform across the cohort (Section 2.5), but no per-patient checklist of candidate precipitants was completed; a trigger that was genuinely absent therefore cannot be distinguished with certainty from one that was present but not specifically sought, and this applies equally to *de novo* and to chronic HF presentations. A recorded result for the protocol-mandated investigations was available in a comparable proportion of each group — natriuretic peptide in 97.2–98.6% (*p* = 0.667), admission eGFR in all 486 patients and echocardiographic RV assessment in 99.6%–100% — so these findings do not suggest lower availability of the recorded investigations in the unknown group; differential ascertainment of specific precipitants nevertheless cannot be excluded. Ambiguous cases with approximately equal behavioral and clinical contribution were assigned to the behavioral group by convention, which may have introduced misclassification affecting the behavioral-versus-clinical comparison; its direction and magnitude are not estimable from the group comparisons reported here. A further consequence of the same convention is that the behavioral group is not composed exclusively of patients with an isolated behavioral trigger: 75 of its 148 patients also had a co-existing clinical trigger that was judged the lesser contributor. The behavioral group is therefore best understood as a predominant-precipitant category rather than as a mutually exclusive trigger type, and the magnitude of the behavioral-versus-clinical contrast, and to a lesser degree of the comparisons that use the behavioral group as reference, depends on this assignment rule. The unknown group is homogeneous by construction, since none of its 108 patients had a precipitating factor of either kind recorded.

All-cause rather than HF-specific mortality and hospitalization were used as endpoints, and cause of death was adjudicated only for in-hospital deaths; the excess mortality of the unknown group may therefore in part reflect competing non-cardiovascular comorbidity. Individual non-fatal outcomes were originally ascertained conditional on survival; although the competing-risk analysis addresses the resulting informative censoring for first AHF rehospitalization, recurrent-event burden beyond the first rehospitalization could not be reconstructed with the same rigor. Chronic HF pharmacotherapy, including loop diuretic use, was recorded at admission and again at discharge, but in-hospital diuretic doses and cumulative loop-diuretic exposure during the index hospitalization were not captured. True diuretic resistance can therefore not be formally distinguished from insufficient decongestive therapy or from persistent congestion as the driver of RRT in this cohort.

CA125 was not measured, precluding assessment of the combined NT-proBNP/CA125 congestion signature, which has been associated with worse prognosis than elevation of either marker alone, and of CA125-guided decongestion strategies (32).

Finally, the unknown group comprised 108 patients from a single center, limiting power for further subgroup analysis. The complementary question of which patient-level features within this phenotype are most prognostically informative would be better addressed by dedicated phenotyping and clustering analyses in larger multicenter cohorts.

## 6 Conclusions

In this prospective cohort of consecutive AHF admissions, patients without an identifiable precipitating factor had substantially worse 12-month survival than those with behavioral or clinical precipitants, in the context of a distinctive profile of advanced frailty, RV dysfunction, cardio-renal impairment and multivalvular disease. The between-group difference appeared to be driven predominantly by mortality rather than by differences in first AHF rehospitalization, and the association was attenuated after adjustment for the variables that define the phenotype.

The absence of an identifiable precipitant may therefore be useful as an early, readily available integrative marker of advanced disease trajectory rather than a documentation failure. This hypothesis requires confirmation in prospective multicenter studies before it can inform management.

## Data Availability

The raw data supporting the conclusions of this article will be made available by the authors, without undue reservation.

## Supplementary material

SUPPLEMENTARY TABLE 1

Echocardiographic classification of pulmonary hypertension probability according to 2022 ESC/ERS Guidelines.

SUPPLEMENTARY TABLE 2

Classification of chronic kidney disease stages according to KDIGO 2012 guidelines.

SUPPLEMENTARY TABLE 3

Clinical Frailty Scale (CFS) categories and descriptions.

SUPPLEMENTARY TABLE 4

Clinical phenotypes of acute heart failure at presentation according to the 2021 ESC Heart Failure Guidelines.

SUPPLEMENTARY TABLE 5

Multivariable Cox proportional hazards models for each endpoint, with and without precipitant category.

SUPPLEMENTARY TABLE 6

Sensitivity analysis restricted to patients with chronic heart failure (de novo presentations excluded).

SUPPLEMENTARY TABLE 7

Competing-risk analysis of first AHF rehospitalization, with death and terminal renal replacement therapy as competing events.

SUPPLEMENTARY TABLE 8

Renal function at baseline and at admission by precipitating factor group.

SUPPLEMENTARY TABLE 9

Sensitivity of the association between precipitant category and all-cause death to the renal variable used in adjustment.

SUPPLEMENTARY FIGURE 1

Kaplan-Meier event-free survival curves for the triple composite endpoint in a sensitivity analysis excluding patients who died during the index hospitalization (n=460).

SUPPLEMENTARY FIGURE 2

Kaplan-Meier overall survival curves for all-cause death after exclusion of patients presenting with de novo heart failure (n=354).

SUPPLEMENTARY FIGURE 3

Cumulative incidence of first AHF rehospitalization, estimated with the Aalen-Johansen method treating death and terminal renal replacement therapy as competing events.
