# Supplementary material for: Beyond the trigger: the unknown precipitant as a high-risk phenotype in acute heart failure
Source: Front Cardiovasc Med. 2026 Oct 30;13:1880740. doi: 10.3389/fcvm.2026.1880740 (PMC13759750; doi:10.3389/fcvm.2026.1880740)
Supplement: Supplementary file 1 [file Datasheet1.docx]

Supplementary Material

# 1 Supplementary Data

**Supplementary Table 1.** Echocardiographic classification of pulmonary hypertension (PH) probability according to 2022 ESC/ERS Guidelines.

| **TR peak velocity (m/s)** | **Additional PH signs*** | **PH probability** |
| --- | --- | --- |
| ≤2.8 or not measurable | No | Low |
| ≤2.8 or not measurable | Yes | Moderate |
| 2.9–3.4 | No | Moderate |
| 2.9–3.4 | Yes | High |
| >3.4 | Not required | High |

* Additional echocardiographic signs of PH: RV/LV basal diameter ratio >1.0; flattening of the interventricular septum (eccentricity index >1.1 in systole and/or diastole); RVOT acceleration time <105 msec and/or mid-systolic notching; early diastolic pulmonary regurgitation velocity >2.2 m/s; pulmonary artery diameter >25 mm; IVC diameter >21 mm with decreased inspiratory collapse (<50% with sniff or <20% with quiet inspiration); RA area >18 cm² in end-systole. TR = tricuspid regurgitation; RV = right ventricle; LV = left ventricle; RVOT = right ventricular outflow tract; IVC = inferior vena cava; RA = right atrium.

**Supplementary Table 2.** Classification of chronic kidney disease stages according to KDIGO 2012 guidelines.

| CKD Stage | eGFR (ml/min/1.73m²) | Description |
| --- | --- | --- |
| G1 | ≥90 | Normal or high |
| G2 | 60–89 | Mildly decreased |
| G3a | 45–59 | Mildly to moderately decreased |
| G3b | 30–44 | Moderately to severely decreased |
| G4 | 15–29 | Severely decreased |
| G5 | <15 | Kidney failure |

eGFR = estimated glomerular filtration rate. CKD was defined as eGFR <60 ml/min/1.73m² persisting for ≥3 months.

**Supplementary Table 3.** Clinical Frailty Scale (CFS) categories and descriptions.

| Score | Category | Description |
| --- | --- | --- |
| 1 | Very Fit | Robust, active, energetic, motivated; regularly exercise and are among the fittest for their age. |
| 2 | Well | No active disease symptoms, less fit than category 1. Often exercise or very active occasionally. |
| 3 | Managing well | Medical problems well-controlled. Not regularly active beyond routine walking. |
| 4 | Vulnerable | Not dependent but symptoms limit activities. Often complain of slowing down. |
| 5 | Mildly frail | Evident slowing. Dependent in instrumental ADLs (finances, transport, heavy housework). |
| 6 | Moderately frail | Need help with all outside activities and with housekeeping. Difficulty with stairs. |
| 7 | Severely frail | Completely dependent for personal care; however, appears stable and not at high risk of dying within 6 months. |
| 8 | Very severely frail | Completely dependent, approaching end of life. Typically, could not recover even from a minor illness. |
| 9 | Terminally ill | Approaching end of life. This category applies even if otherwise not evidently frail. |

Study classification: Non-frail (CFS 1–3); Pre-frail/Mildly frail (CFS 4–5); Frail (CFS 6–9). ADLs = activities of daily living.

**Supplementary Table 4.** Clinical phenotypes of acute heart failure at presentation according to the 2021 ESC Heart Failure Guidelines.

| Phenotype | Key Clinical Features |
| --- | --- |
| Acute Decompensated HF (ADHF) | Gradual worsening of pre-existing HF symptoms (dyspnea, edema). Hemodynamically stable. No signs of hypoperfusion. Most common presentation (~75% of AHF). |
| Acute Pulmonary Edema (APO) | Severe respiratory distress, orthopnea, low oxygen saturation, bilateral crackles/wheeze. Rapid onset. Evidence of pulmonary congestion on imaging. |
| Isolated Right Heart Failure (Isolated RHF) | Predominant right-sided congestion (elevated JVP, peripheral edema, hepatomegaly) without significant left-sided congestion or LV systolic dysfunction (LVEF ≥40%). Reduced RV systolic function (TAPSE <17 mm, TV S′ <9.5 cm/s). |
| Cardiogenic shock (CS) | Hypotension (SBP <90 mmHg for >30 min or vasopressors required) with evidence of end-organ hypoperfusion (oliguria, elevated lactate, cold peripheries, altered mental status) despite adequate filling. |

HF = heart failure; JVP = jugular venous pressure; LV = left ventricle; LVEF = left ventricular ejection fraction; RV = right ventricle; TAPSE = tricuspid annular plane systolic excursion; TV S′ = tricuspid lateral annular systolic velocity; SBP = systolic blood pressure.

# 2 Supplementary Figures and Tables

## 2.1 Supplementary Figures

**Supplementary Figure 1.** Kaplan-Meier event-free survival curves for the triple composite endpoint (all-cause death, AHF rehospitalization or renal replacement therapy) after exclusion of patients who died during the index hospitalization (n=460). Event-free survival at 12 months was 59.6% in the behavioral, 56.3% in the non-behavioral/clinical and 38.4% in the unknown group. Overall log-rank p=0.002; pairwise behavioral vs. non-behavioral p=0.547, behavioral vs. unknown p<0.001, non-behavioral vs. unknown p=0.003. The separation persisted after discharge and was not explained solely by in-hospital mortality. Shaded areas represent 95% confidence intervals.

**Supplementary Figure 2**. Kaplan-Meier overall survival curves for all-cause death (primary endpoint) after exclusion of patients presenting with de novo heart failure (chronic HF only, n=354). Survival at 12 months was 76.5% in the behavioral, 63.0% in the non-behavioral/clinical and 51.6% in the unknown group. Overall log-rank p<0.001; pairwise behavioral vs. non-behavioral p=0.021, behavioral vs. unknown p<0.001, non-behavioral vs. unknown p=0.052. Shaded areas represent 95% confidence intervals.

**Supplementary Figure 3.** Cumulative incidence of first AHF rehospitalization, estimated with the Aalen–Johansen method treating death and terminal renal replacement therapy as competing events (solid lines), together with the cumulative incidence of the competing event itself (dashed lines), stratified by precipitating factor group. Between-group comparison by Gray’s test p=0.207. Shaded areas represent 95% confidence intervals.

**Supplementary Table 5.** Multivariable Cox proportional hazards models for each endpoint, with and without precipitant category (n=475 with complete covariate data).

| **Variable** | **All-cause death HR (95% CI)** | **p** | **Death/RRT HR (95% CI)** | **p** | **Triple composite HR (95% CI)** | **p** |
| --- | --- | --- | --- | --- | --- | --- |
| Age (per year) | 1.02 (0.99–1.05) | 0.131 | 1.00  (0.98–1.03) | 0.735 | 1.00  (0.98–1.02) | 0.727 |
| Admission eGFR (per ml/min/1.73m²) | 0.99 (0.98–1.00) | 0.061 | 0.98 (0.97–0.99) | 0.001 | 0.99  (0.98–0.99) | 0.001 |
| Right heart failure | 1.91 (1.33–2.74) | <0.001 | 1.89  (1.33–2.69) | <0.001 | 1.36  (1.03–1.79) | 0.029 |
| log NT-proBNP | 1.63 (1.31–2.03) | <0.001 | 1.54  (1.24–1.90) | <0.001 | 1.24  (1.05–1.45) | 0.010 |
| Frailty (CFS ≥5) | 1.75 (1.01–3.03) | 0.047 | 1.87  (1.09–3.20) | 0.023 | 2.26  (1.50–3.41) | <0.001 |
| Non-behavioral/clinical vs. behavioral | 1.65 (1.07–2.55) | 0.024 | 1.85  (1.20–2.85) | 0.005 | 1.31  (0.96–1.80) | 0.092 |
| Unknown vs. behavioral | 1.43 (0.88–2.31) | 0.150 | 1.55  (0.97–2.50) | 0.069 | 1.33  (0.93–1.91) | 0.117 |
| Likelihood ratio test for precipitant category | χ²=5.43, df=2 | 0.066 | χ²=8.46, df=2 | 0.015 | χ²=3.60, df=2 | 0.165 |
| C-index without / with precipitant category | 0.716 / 0.724 | — | 0.727 / 0.737 | — | 0.679 / 0.683 | — |

*CI = confidence interval; CFS = Clinical Frailty Scale; eGFR = estimated glomerular filtration rate; HR = hazard ratio; RRT = renal replacement therapy. The proportional hazards assumption was satisfied in all models. The likelihood ratio test compares nested models with and without precipitant category and tests whether the categorization carries prognostic information incremental to the phenotype-defining covariates.*

**Supplementary Table 6**. Sensitivity analysis restricted to patients with chronic heart failure (de novo presentations excluded; n=354).

| **Endpoint** | **Behavioral (n=115)** | **Non-behavioral/ clinical (n=146)** | **Unknown (n=93)** | **Overall log-rank p** |
| --- | --- | --- | --- | --- |
| All-cause death — 12-month survival | 76.5% | 63.0% | 51.6% | <0.001 |
| All-cause death — HR vs. behavioral | reference | 1.71 (1.08–2.71) | 2.53 (1.57–4.07) | — |
| Death/RRT — HR vs. behavioral | reference | 1.89 (1.20–2.98) | 2.80 (1.75–4.49) | <0.001 |
| Triple composite — HR vs. behavioral | reference | 1.52 (1.08–2.13) | 2.03 (1.42–2.91) | <0.001 |
| Pairwise p — behavioral vs. non-behavioral | — | death 0.021; death/RRT 0.005; composite 0.016 | — | — |
| Pairwise p — non-behavioral vs. unknown | — | — | death 0.052; death/RRT 0.045; composite 0.073 | — |

*HR = hazard ratio; RRT = renal replacement therapy. Hazard ratios are unadjusted, with the behavioral group as reference. In the full cohort the corresponding hazard ratios for the unknown group were 2.44 (all-cause death), 2.69 (death/RRT) and 1.96 (triple composite).*

**Supplementary Table 7.** Competing-risk analysis of first AHF rehospitalization, with death and terminal renal replacement therapy as competing events.

|  | **Behavioral (n=148)** | **Non-behavioral/ clinical (n=230)** | **Unknown (n=108)** |
| --- | --- | --- | --- |
| First AHF rehospitalizations (n) | 44 | 60 | 39 |
| Cumulative incidence at 3 months | 14.9% | 13.9% | 16.7% |
| Cumulative incidence at 6 months | 20.9% | 20.4% | 24.1% |
| Cumulative incidence at 12 months (95% CI) | 29.7% (22.6–37.2) | 26.1% (20.6–31.9) | 36.1% (27.1–45.1) |
| Cumulative incidence of competing event at 12 months | 11.5% | 21.3% | 28.7% |
| Fine–Gray sHR vs. behavioral | reference | 0.87 (0.59–1.29) | 1.25 (0.82–1.93) |

*CI = confidence interval; sHR = subdistribution hazard ratio. Cumulative incidence estimated by the Aalen–Johansen method. Overall between-group comparison by Gray’s test: p=0.207 (pairwise behavioral vs. non-behavioral p=0.498, behavioral vs. unknown p=0.296, non-behavioral vs. unknown p=0.077). This analysis recovered 53 first AHF rehospitalizations absent from the survivor-conditional follow-up field because the patient had by then reached a competing event and left the survivor-based denominator (51 death, two terminal RRT).*

**Supplementary Table 8**. Renal function at baseline and at admission, stratified by precipitating factor group (n=486).

| **Variable** | **Behavioral (n=148)** | **Non-behavioral/clinical (n=230)** | **Unknown (n=108)** | **p** |
| --- | --- | --- | --- | --- |
| Baseline eGFR (ml/min/1.73 m²) | 62 [45.8–77] | 62.5 [47–78] | 48 [36–65.2] | <0.001 |
| Admission eGFR (ml/min/1.73 m²) | 60.5 [42.8–79.5] | 58 [45–77] | 44 [29–64.2] | <0.001 |
| Change in eGFR, admission − baseline (ml/min/1.73 m²) | 0 [−4.2 to 4] | −1 [−6 to 2.8] | −1 [−9 to 2] | 0.083 |
| Baseline creatinine (mg/dl) | 1.1 [0.9–1.3] | 1.1 [0.9–1.4] | 1.3 [1.0–1.7] | <0.001 |
| Baseline CKD stage 3B–4, n (%) | 35 (23.6%) | 52 (22.6%) | 50 (46.3%) | <0.001 |
| Acute kidney injury during admission (KDIGO ≥1), n (%) | 62 (41.9%) | 107 (46.5%) | 70 (64.8%) | <0.001 |

*CKD = chronic kidney disease; eGFR = estimated glomerular filtration rate; KDIGO = Kidney Disease: Improving Global Outcomes. Continuous variables are presented as median [25th–75th percentile] and compared by the Kruskal–Wallis test; categorical variables are presented as n (%) and compared by the chi-square test. Baseline eGFR and creatinine were derived as defined in Section 2.3 and were available for all 486 patients. Change in eGFR is the difference between the admission and baseline values, with negative values denoting a fall at presentation. Baseline CKD stage 3B–4 corresponds to a baseline eGFR <45 ml/min/1.73 m². Acute kidney injury during the index hospitalization was defined by the KDIGO 2012 serum creatinine criteria — an increase of ≥0.3 mg/dl, or a rise to ≥1.5 times the baseline value — applied to the peak in-hospital creatinine relative to the baseline value defined in Section 2.3, and staged accordingly; urine output criteria were not applied. The p value shown is for the dichotomous comparison (any acute kidney injury versus none); the full four-level stage distribution also differed between groups (chi-square p=0.005), as did the full distribution of baseline CKD stage (chi-square p<0.001).*

**Supplementary Table 9**. Sensitivity of the association between precipitant category and all-cause death to the renal variable used in adjustment.

| **Model** | **Non-behavioral/clinical vs. behavioral, HR (95% CI)** | **Unknown vs. behavioral, HR (95% CI)** | **Likelihood ratio test for precipitant category, p** |
| --- | --- | --- | --- |
| A. Unadjusted and renal-adjusted Cox models |  |  |  |
| Unadjusted | 1.55 (1.01–2.38) | 2.44 (1.54–3.88) | — |
| + admission eGFR | 1.52 (0.99–2.34) | 1.85 (1.16–2.96) | — |
| + baseline eGFR | 1.58 (1.03–2.43) | 2.04 (1.28–3.26) | — |
| + baseline CKD stage 3B–4 | 1.57 (1.02–2.41) | 2.09 (1.31–3.34) | — |
| + admission and baseline eGFR | 1.49 (0.97–2.29) | 1.85 (1.15–2.96) | — |
| B. Full multivariable models (n=475) |  |  |  |
| with admission eGFR | 1.65 (1.07–2.55) | 1.43 (0.88–2.31) | 0.066 |
| with baseline eGFR | 1.68 (1.09–2.59) | 1.50 (0.93–2.42) | 0.050 |
| with baseline CKD stage 3B–4 | 1.67 (1.08–2.57) | 1.50 (0.92–2.43) | 0.055 |
| with admission and baseline eGFR | 1.57 (1.02–2.44) | 1.40 (0.86–2.27) | 0.111 |

*CI = confidence interval; CKD = chronic kidney disease; eGFR = estimated glomerular filtration rate; HR = hazard ratio. All models are Cox proportional hazards models for all-cause death at 12 months with the behavioral group as reference. Panel A: unadjusted model and models adjusted only for the renal variable shown (n=486). Panel B: multivariable models additionally including age, right heart failure, log NT-proBNP and frailty (CFS ≥5), fitted in the 475 patients with complete covariate data; the likelihood ratio test compares each model with and without precipitant category (2 degrees of freedom); its exact value for the baseline-eGFR model was 0.0502. The corresponding C-indices were 0.724, 0.722, 0.724 and 0.725. Considered alone, admission eGFR discriminated 12-month mortality better than baseline eGFR (C-index 0.658 vs. 0.617); in a model containing the two renal measures alone, without precipitant category, only admission eGFR remained independently associated with mortality (p<0.001 vs. p=0.100), and in the corresponding model that also included precipitant category baseline eGFR was likewise non-significant (p=0.115). The proportional hazards assumption was satisfied for the models using admission eGFR and baseline CKD stage, but was violated for baseline eGFR modeled as a continuous variable (Schoenfeld p=0.01); the categorical CKD-stage model is therefore the preferred alternative parameterization.*
